# Supplementary material for: Exploring BenzylethoxyAryl Urea Scaffolds for Multitarget Immunomodulation Therapies
Source: Int J Mol Sci. 2023 May 11;24(10):8582. doi: 10.3390/ijms24108582 (PMC10218498; doi:10.3390/ijms24108582)
Supplement: Supplementary file 1 [file ijms-24-08582-s001.zip › ijms-2344383-supplementary.pdf]

Exploring BenzylethoxyAryl-Ureas Scaffolds for Multitarget Immunomodulation Therapies

Raquel Gil-Edo [1], German Hernández-Ribelles [2], Santiago Royo [3], Natasha Thawait [4], Alan Serrels [4], Miguel Carda [1] and Eva Falomir [1,*]

1 Inorganic and Organic Chemistry Department, University Jaume I, 12071 Castellón, Spain; ragil@uji.es (R.G.-E.)
2 Curapath, Benjamin Franklin Avenue 19, 46980 Paterna, Spain
3 Institute of Agronomic Engineering for Development, Polytechnic University of Valencia, 46022 Valencia, Spain
4 Cancer Research UK Scotland Centre, Institute of Genetics and Cancer, University of Edinburgh, Crewe Road South, Edinburgh EH4 2XR, UK
* Correspondence: efalomir@uji.es

Supporting Information

Contents:

Suplementary information .... - 1 -
Experimental procedure .... - 3 -
Analytical NMR spectra .... - 4 -
Graphical NMR spectra .... - 13 -

Suplementary information

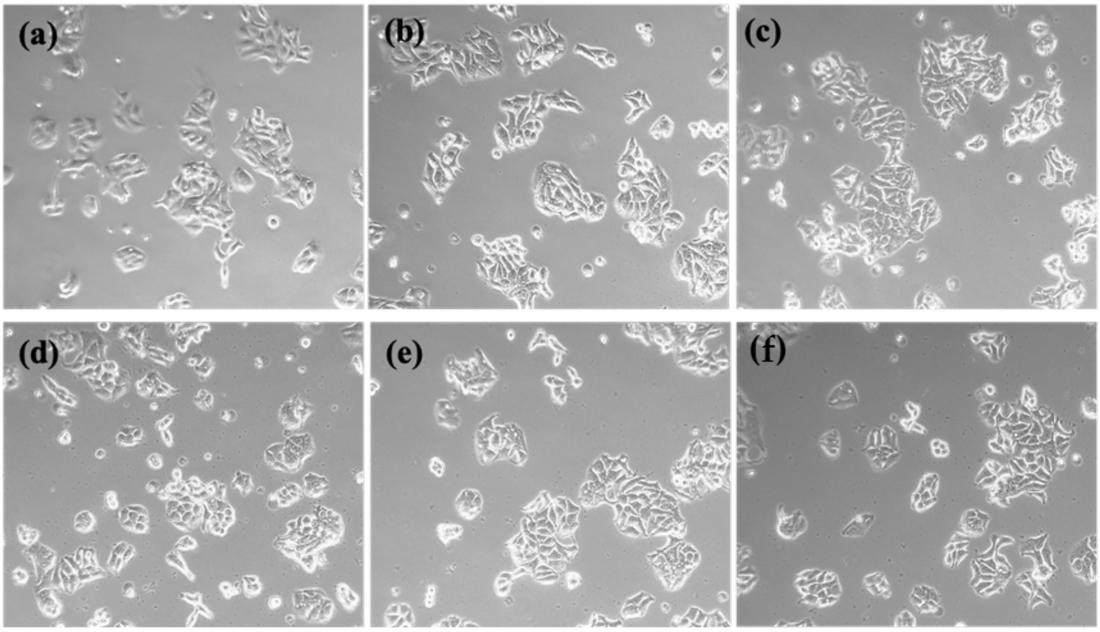

Figure S1. Morphological control of HT29 cells after 48 hours of exposure to compounds with no THP-1 cells. (a) Control; (b) **BMS-8** at 20 μM; (c) **14** at 20 μM; (d) **2** at 20 μM; (e) **8** at 20 μM; (f) **11** at 20 μM. (magnifications.×20).

Table S1. Antibody details for the *ex vivo* study of CTLs exhaustion receptors by flow cytometry.

**Marker**	**Fluorophore**	**Source**
**CD8**	PE	eBioscience
**CD45**	AF488	eBioscience
**CD69**	E450	eBioscience
**LAG-3**	BV711	BD Bioscience
**OX-40**	PerCP/Cy5.5	BioLegend
**TIM-3**	PE-Dazzle	BioLegend
**PD-1**	APC	Invitrogen
**Viability**	Zombie NIR	BioLegend

Table S2. Effect of the tested compounds on the most proximal molecules to the TCRs, Fyn and Lck, together with its activated forms PhosphoY394-Lck and PhosphoY505-Lck

Table S2. Initial signaling protein expression promoted by TCRs inside OT-1

Comp.	LCK	pY394-LCK	pY505-LCK	FYN
**2**	176	143	140	101
**9**	180	175	154	108
**11**	168	184	157	109
**14**	175	130	145	102

Table S3. Effect on the downstream signaling molecules generated from the activation of Lck, such as the tyrosine kinases ZAP70 and LAT.

Table S3. Signaling protein expression promoted by TCRs inside OT-1

Comp.	ZAP-70	pZAP-70	LAT	pLAT
**2**	34	88	63	9
**8**	37	74	88	8
**11**	38	77	85	10
**14**	54	108	103	21

Experimental procedure

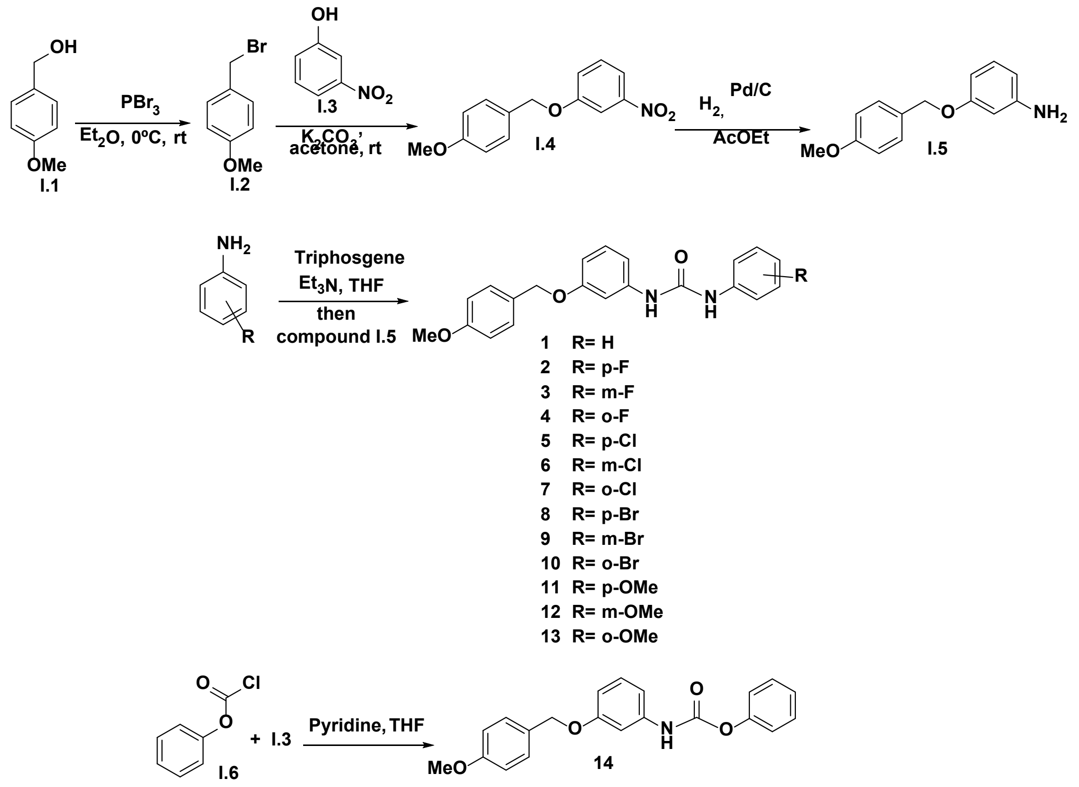

1-(bromomethyl)-4-methoxybenzene (I.2)

A solution of 759 µL of PBr_3 (7.83 mmol) in Et_2O (15.7 mL, 2 mL/mmol PBr_3) was added dropwise, at 0 ºC under a N_2 atmosphere, to a solution of (4-nethoxyphenyl)methanol **(I.1)** (2.05 g, 14.51 mmol) in Et_2O (2.9 mL). The mixture was stirred at room temperature for 2 hr. Then, the solution was poured into ice and the organic layer was separated and treated with and aqueous saturated solution of $NaHCO_3$, washed with brine and dried over Na_2SO_4. Evaporation of the solvent afforded compound **I.2** (12.8 g, 96%) as a pale yellow oil which was used directly without further purification for the following reaction.

1-((4-methoxybenzyl)oxy)-3-nitrobenzene (I.4)

A mixture of 1-(bromomethyl)-4-methoxybenzene **(I.2)** (2.8 g, 13.93 mmol), 3-nitrophenol **(I.3)** **(**3.53 g, 17.41 mmol) and K_2CO_3 (2.9 g, 20.89 mmol) in acetone (52 mL) was stirred for 16 h at room temperature. After removal of the solvent, the residue was taken up in ethyl acetate, washed with aqueous 1M NaOH solution and brine and dried over Na_2SO_4. After filtration and removal of the solvent compound **I.5** (3.25 g, 90%) was obtained as crystalline yellow solid.

3-((4-methoxybenzyl)oxy)aniline (I.5)

A solution of 1-((4-methoxybenzyl)oxy)-3-nitrobenzene **(I.4)** (1.72 g, 6.61 mmol) in AcOEt (40 mL) was added to AcOEt (6.85 mL) containing Pd/C (10%) (343 mg, 20 % by weight). The resulting mixture was stirred overnight under H_2 atmosphere and then filtered through Celite, yielding 1.27 g (84%) of compound **I.5** as a brown solid.

Synthesis of (phenyl (3-((4-methoxybenzyl)oxy)phenyl)carbamate) (14)

A solution of 3-((4-methoxybenzyl)oxy)aniline **(I.5)** (407 mg, 1.78 mmol) in dry THF (8.0 mL) was cooled at 0 °C and then anhydrous pyridine (357 µL, 4.44 mmol) and phenyl chloroformiate **(I.6)** (344 µL, 2.66 mmol) were added under nitrogen atmosphere. The resulting mixture was stirred for 20 min at 0°C and at room temperature for 1 h. Then, water (8.88 mL) and 1M aqueous HCl (4.44 mL) were added and the mixture was extracted with CH_2Cl_2 (3 x 20 mL). The combined organic phases were washed with brine and dried over Na_2SO_4. The solvent was removed *in vacuo* and the residue was purified by silica gel chromatography using hexane: ethyl acetate (95:5) as eluent yielding 483 mg (78 %) of carbamate **I.4** as a white solid.

Analytical NMR spectra

1-(bromomethyl)-4-methoxybenzene (I.2): Yield 96%; yellow oil; 1H NMR (400 MHz, $CDCl_3$); δ 7.34 (d, J = 8.8 Hz, 2H), 6.88 (d, J = 8.7 Hz, 2H), 4.52 (s, 2H), 3.82 (s, 3H).; 13C NMR (100 MHz, $CDCl_3$) δ 159.66 (C), 130.39 (2CH), 129.93 (C), 114.19 (2CH), 55.28 (CH2), 33.90 (CH3).

1-((4-methoxybenzyl)oxy)-3-nitrobenzene (I.4): Yield 90%; yellow solid; m.p.80-84°C; 1H NMR (400 MHz, $CDCl_3$); δ 7.76 – 7.69 (m, 2H), 7.37 – 7.30 (m, 1H), 7.28 (d,

J = 8.7 Hz, 2H), 7.21 – 7.17 (m, 1H), 6.85 (d, J = 8.7 Hz, 2H), 4.98 (s, 2H), 3.74 (s, 3H).; 13C NMR (100 MHz, $CDCl_3$) δ 159.79 (C), 159.27 (C), 149.22 (C), 129.91 (CH), 129.36 (2CH), 127.73 (C), 122.01 (CH), 115.89 (CH), 114.18 (2CH), 109.22 (CH), 70.46 (CH2), 55.31 (CH3).

1-((4-methoxybenzyl)oxy)aniline (I.5): Yield 84%; brown solid; m.p.106-108ºC; 1H NMR (400 MHz, $CDCl_3$); δ 7.26 (d, J = 8.7 Hz, 2H), 6.97 (t, J = 8.0 Hz, 1H), 6.83 (d, J = 8.7 Hz, 2H), 6.31 (ddd, J = 8.2, 2.3, 0.9 Hz, 1H), 6.26 – 6.19 (m, 2H), 4.86 (s, 2H), 3.73 (s, 3H), 3.39 (s, 2H).; 13C NMR (100 MHz, $CDCl_3$) δ 160.07 (C), 159.39 (C), 147.71 (C), 130.07 (CH), 129.27 (C), 129.14 (2CH), 113.97 (2CH), 108.11 (CH), 104.92 (CH), 102.08 (CH), 69.60 (CH2), 55.28 (C3); HR ESMS *m/z* 230.1174 [M-H]+. Calc. for $C_{14}H_{15}N_1O_2$ 230.1181.

1-(3-((4-methoxybenzyl)oxy)phenyl)-3-phenylurea (1): Yield 95%; white solid; m.p.199-200ºC; 1H NMR (400 MHz, DMSO); δ 8.63 (s, 2H), 7.44 (dd, J = 8.6, 1.1 Hz, 2H), 7.38 (d, J = 8.7 Hz, 2H), 7.31 – 7.24 (m, 2H), 7.23 (t, J = 2.2 Hz, 1H), 7.16 (t, J = 8.1 Hz, 1H), 7.00 – 6.91 (m, 4H), 6.61 (dd, J = 8.2, 2.5 Hz, 1H), 4.99 (s, 2H), 3.76 (s, 3H).; 13C NMR (100 MHz, DMSO) δ 158.93 (C), 158.81 (C), 152.40 (C), 140.86 (C), 139.59 (2CH), 129.46 (CH), 129.35 (2CH), 128.96 (C), 128.72 (2CH), 121.80 (CH), 118.19 (2CH), 113.77 (2CH), 110.64 (CH), 108.06 (CH), 104.88 (CH), 68.83 (CH2), 55.06 (CH3); IR $_{vmax}$ (cm-1) 3296 (N-H), 1633 (C=O), 1558 (N-H amide), 1515, 1489 (N-C=O), 1249,1176,1028 (C-O).; HR ESMS *m/z* 349.1552 [M-Na]+. Calc. for $C_{21}H_{20}N_2O_3$ 349.1552.

1-(4-fluorophenyl)-3-(3-((4-methoxybenzyl)oxy)phenyl)urea (2): Yield 61%; white solid; m.p.201-205ºC; 1H NMR (400 MHz, DMSO); δ 8.68 (s, 1H), 8.64 (s, 1H), 7.45 (dd, J = 9.2, 4.9 Hz, 2H), 7.37 (d, J = 8.7 Hz, 2H), 7.22 (t, J = 2.2 Hz, 1H), 7.16 (t, J = 8.1 Hz, 1H), 7.11 (t, J = 8.9 Hz, 2H), 6.97 – 6.90 (m, 3H), 6.61 (ddd, J = 8.2, 2.5, 0.8 Hz, 1H), 4.98 (s, 2H), 3.76 (s, 3H).; 13C NMR (100 MHz, DMSO) δ 158.93 (C), 158.81 (C), 152.40 (C), 140.86 (C), 139.59 (2CH), 129.46 (CH), 129.35 (2CH), 128.96 (C), 128.72 (2CH), 121.80 (CH), 118.19 (2CH), 113.77 (2CH), 110.64 (CH), 108.06 (CH), 104.88 (CH), 68.83 (CH2), 55.06 (CH3); ^{19}F NMR (377 MHz, DMSO) δ -121.49.; IR $_{vmax}$ (cm-1) 3292 (N-H), 1633 (C=O), 1563 (N-H amide), 1508 (N-C=O), 1249,1172,1009 (C-O).; HR ESMS *m/z* 367.1457 [M-H]+. Calc. for $C_{21}H_{19}N_2O_3F$ 367.1458.

1-(3-fluorophenyl)-3-(3-((4-methoxybenzyl)oxy)phenyl)urea (3): Yield 80%; white solid; m.p.202-203ºC; 1H NMR (400 MHz, DMSO); δ 8.90 (s, 1H), 8.73 (s, 1H), 7.48 (dt, J = 12.0, 2.3 Hz, 1H), 7.38 (d, J = 8.7 Hz, 2H), 7.30 (td, J = 8.2, 7.0 Hz, 1H), 7.22 (t, J = 2.2 Hz, 1H), 7.17 (t, J = 8.1 Hz, 1H), 7.11 (dd, J = 8.2, 1.1 Hz, 1H), 6.95 (d, J = 8.7 Hz, 3H), 6.77 (td, J = 8.5, 2.6 Hz, 1H), 6.63 (dd, J = 8.1, 2.4 Hz, 1H), 4.99 (s, 2H), 3.76 (s, 3H).; 13C NMR (100 MHz, DMSO)) δ 162.36 (C, d, J = 240.6 Hz), 158.94 (C), 158.80 (C), 152.25 (C), 141.50 (C, d, J = 11.5 Hz), 140.57 (C), 130.25 (d, J = 9.9 Hz), 129.49 (CH), 129.36 (2CH), 128.94 (C), 113.90 (CH, d, J = 2.4 Hz), 113.77 (2CH), 110.80 (CH), 108.32 (CH), 108.10 (CH, d, J = 21.2 Hz), 105.07 (CH), 104.83 (CH, d, J = 26.4 Hz), 68.85 (CH2), 55.06 (CH3); ^{19}F NMR (377 MHz, DMSO) δ -112.29.; IR $_{vmax}$ (cm-1) 3292 (N-H), 1630 (C=O), 1558 (N-H amide), 1508 (N-C=O), 1244,1176,1009 (C-O).; HR ESMS *m/z* 367.1457 [M-H]+. Calc. for $C_{21}H_{19}N_2O_3F$ 367.1458.

1-(2-fluorophenyl)-3-(3-((4-methoxybenzyl)oxy)phenyl)urea (4): Yield 41%; white solid; m.p.185-186ºC; 1H NMR (400 MHz, DMSO); δ 9.04 (s, 1H), 8.52 (d, J = 2.2 Hz, 1H), 8.14 (td, J = 8.3, 1.5 Hz, 1H), 7.38 (d, J = 8.5 Hz, 2H), 7.27 – 7.20 (m, 2H), 7.20 – 7.16 (m, 1H), 7.13 (t, J = 7.9 Hz, 1H), 7.04 – 6.97 (m, 1H), 6.95 (d, J = 8.5 Hz, 2H), 6.90 – 6.93 (m, 1H), 6.64 (dd, J = 8.2, 2.4 Hz, 1H), 4.99 (s, 2H), 3.76 (s, 3H).; 13C NMR (100 MHz, DMSO)) δ 158.94 (C), 158.85 (C), 152.06 (C), 151.99 (C, d, J = 241.2 Hz), 140.58 (C), 129.56 (CH), 129.35 (2CH), 128.93 (C), 127.45 (CH, d, J = 10.3 Hz), 124.45 (CH, d, J = 3.4 Hz), 122.44 (CH, d, J = 7.4 Hz), 120.58 (CH), 114.90 (CH, d, J = 19.1 Hz), 113.78 (CH), 110.56 (CH), 108.34 (CH), 104.81 (CH), 68.84 (CH2), 55.06 (CH3); ^{19}F NMR (377 MHz, DMSO) δ -129.97.; IR $_{vmax}$ (cm-1) 3292 (N-H), 1633 (C=O), 1563 (N-H amide), 1508 (N-C=O), 1249,1172,1009 (C-O).; HR ESMS *m/z* 367.1457 [M-H]+. Calc. for $C_{21}H_{19}N_2O_3F$ 367.1458.

1-(4-chlorophenyl)-3-(3-((4-methoxybenzyl)oxy)phenyl)urea (5): Yield 90%; white solid; m.p. 213-215ºC; 1H NMR (400 MHz, DMSO); δ 8.80 (s, 1H), 8.69 (s, 1H), 7.48 (d, J = 8.9 Hz, 2H), 7.38 (d, J = 8.7 Hz, 2H), 7.32 (d, J = 8.9 Hz, 2H), 7.22 (t, J = 2.2 Hz, 1H), 7.17 (t, J = 8.1 Hz, 1H), 6.98 – 6.90 (m, 3H), 6.62 (dd, J = 8.2, 2.4 Hz, 1H), 4.98 (s, 2H), 3.75 (s, 3H).; 13C NMR (100 MHz, DMSO)) δ 158.96 (C), 158.82 (C), 152.34 (C), 140.71 (C), 138.66 (C), 129.53 CHs), 129.43 (2CH), 128.95 (C), 128.61 (2CH), 125.34

(C), 119.72 (2CH), 113.79 (2CH), 110.76 (CH), 108.22 (CH), 104.98 (CH), 68.84 (CH2), 55.09 (CH3).; IR $_{vmax}$ (cm-1) 3292 (N-H), 1627 (C=O), 1561 (N-H amide), 1512 (N-C=O), 1249,1173,1009 (C-O).; HR ESMS *m/z* 383.1163 [M-H]+. Calc. for $C_{21}H_{19}N_2O_3Cl$ 383.1162.

1-(3-chlorophenyl)-3-(3-((4-methoxybenzyl)oxy)phenyl)urea (6): Yield 80%; white solid; m.p. 203-205ºC; 1H NMR (400 MHz, DMSO); δ 8.91 (s, 1H), 8.77 (s, 1H), 7.71 (t, J = 1.9 Hz, 1H), 7.38 (d, J = 8.7 Hz, 2H), 7.33 – 7.27 (m, 1H), 7.27 – 7.25 (m, 1H), 7.23 (t, J = 2.2 Hz, 1H), 7.17 (t, J = 8.1 Hz, 1H), 7.01 (ddd, J = 7.6, 2.0, 1.4 Hz, 1H), 6.95 (d, J = 8.7 Hz, 3H), 6.63 (dd, J = 8.2, 2.4 Hz, 1H), 4.99 (s, 2H), 3.76 (s, 3H).; 13C NMR (100 MHz, DMSO)) δ 158.93 (C), 158.80 (C), 152.26 (C), 141.20 (C), 140.57 (C), 133.14 (C), 130.32 (CH), 129.49 (CH), 129.36 (2CH), 128.94 (C), 121.40 (CH), 117.53 (CH), 116.62 (CH), 113.77 (2CH), 110.82 (CH), 108.35 (CH), 105.08 (CH), 68.85 (CH2), 55.06 (CH3).; IR $_{vmax}$ (cm-1) 3309 (N-H), 1643 (C=O), 1551 (N-H amide), 1515 (N-C=O), 1249,1182,1009 (C-O).; HR ESMS *m/z* 383.1162 [M-H]+. Calc. for $C_{21}H_{19}N_2O_3Cl$ 383.1162.

1-(2-chlorophenyl)-3-(3-((4-methoxybenzyl)oxy)phenyl)urea (7): Yield 44%; white solid; m.p. 183.4ºC; 1H NMR (400 MHz, DMSO); δ 9.38 (s, 1H), 8.29 (s, 1H), 8.15 (dd, J = 8.3, 1.5 Hz, 1H), 7.45 (dd, J = 8.0, 1.4 Hz, 1H), 7.38 (d, J = 8.7 Hz, 2H), 7.30 (td, J = 7.7, 1.3 Hz, 1H), 7.25 (t, J = 2.2 Hz, 1H), 7.19 (t, J = 8.1 Hz, 1H), 7.03 (ddd, J = 8.0, 7.5, 1.6 Hz, 1H), 6.99 – 6.89 (m, 3H), 6.64 (dd, J = 8.2, 2.4 Hz, 1H), 5.00 (s, 2H), 3.76 (s, 3H).; 13C NMR (100 MHz, DMSO)) δ 158.94 (C), 158.85 (C), 140.59 (C), 135.87 (C), 129.58 (C), 129.34 (2CH), 129.15 (CH), 128.93 (CH), 127.52 (CH), 123.29 (CH), 121.94 (C), 121.35 (CH), 113.78 (2CH), 110.64 (CH), 108.46 (CH), 104.88 (CH), 68.84 (CH2), 55.06 (CH3).; IR $_{vmax}$ (cm-1) 3292 (N-H), 1640 (C=O), 1554 (N-H amide), 1515 (N-C=O), 1249,1186,1014 (C-O).; HR ESMS *m/z* 383.1164 [M-H]+. Calc. for $C_{21}H_{19}N_2O_3Cl$ 383.1162.

1-(4-bromophenyl)-3-(3-((4-methoxybenzyl)oxy)phenyl)urea (8): Yield 80%; white solid; m.p. 224.3ºC; 1H NMR (400 MHz, DMSO); δ 8.77 (s, 1H), 8.66 (s, 1H), 7.50 – 7.39 (m, 4H), 7.37 (d, *J* = 8.7 Hz, 2H), 7.22 (t, *J* = 2.2 Hz, 1H), 7.17 (t, *J* = 8.1 Hz, 1H), 6.99 – 6.89 (m, 3H), 6.63 (dd, *J* = 8.2, 2.4 Hz, 1H), 4.99 (s, 2H), 3.76 (s, 3H).; 13C NMR

(100 MHz, DMSO) δ 158.93 (C), 158.79 (C), 152.26 (C) , 140.65 (C), 139.05 (C), 131.45 (2CH), 129.48 (C), 129.36 (2CH), 128.94 (C), 120.11 (2CH), 113.77 (2CH), 113.17 (CH), 110.75 (CH), 108.25 (CH), 105.00 (CH), 68.83 (CH2), 55.06 (CH3).; IR $_{vmax}$ (cm-1) 3289 (N-H), 1630 (C=O), 1587 (N-H amide), 1518 (N-C=O), 1249,1176,1005 (C-O).; HR ESMS *m/z* 427.0655 [M-H]+. Calc. for $C_{21}H_{19}N_2O_3Br$ 427.0657.

1-(3-bromophenyl)-3-(3-((4-methoxybenzyl)oxy)phenyl)urea (9): Yield 96%; white solid; m.p. 198.5ºC; 1H NMR (400 MHz, DMSO); δ 8.83 (s, 1H), 8.70 (s, 1H), 7.85 (t, J = 1.9 Hz, 1H), 7.38 (d, J = 8.7 Hz, 2H), 7.29 (ddd, J = 8.1, 1.9, 1.2 Hz, 1H), 7.26 – 7.20 (m, 2H), 7.17 (t, J = 7.1 Hz, 1H), 7.16 – 7.12 (m, 1H), 6.98 – 6.91 (m, 3H), 6.63 (dd, J = 8.2, 2.4 Hz, 1H), 4.99 (s, 2H), 3.76 (s, 3H).; 13C NMR (100 MHz, DMSO)) δ 158.93 (C), 158.80 (C), 152.23 (C), 141.32 (C), 140.55 (C), 130.64 (CH), 129.49 (CH), 129.35 (2CH), 128.93 (C), 124.33 (CH), 121.67 (C), 120.39 (CH), 117.02 (CH), 113.76 (2CH), 110.83 (CH), 108.37 (CH), 105.08 (CH), 68.85 (CH2), 55.06 (CH3).; IR $_{vmax}$ (cm-1) 3305 (N-H), 1647 (C=O), 1551 (N-H amide), 1518 (N-C=O), 1248,1182,1009 (C-O).; HR ESMS *m/z* 427.0654 [M-H]+. Calc. for $C_{21}H_{19}N_2O_3Br$ 427.0657.

1-(2-bromophenyl)-3-(3-((4-methoxybenzyl)oxy)phenyl)urea (10): Yield 92%; white solid; m.p. 198.5ºC; 1H NMR (400 MHz, DMSO); δ 9.43 (s, 1H), 8.11 (s, 1H), 8.06 (dd, J = 8.3, 1.5 Hz, 1H), 7.61 (dd, J = 8.0, 1.5 Hz, 1H), 7.38 (d, J = 8.7 Hz, 2H), 7.36 – 7.30 (m, 1H), 7.25 (t, J = 2.2 Hz, 1H), 7.19 (t, J = 8.1 Hz, 1H), 7.01 – 6.97 (m, 1H), 6.95 (d, J = 8.8 Hz, 3H), 6.64 (dd, J = 8.2, 2.4 Hz, 1H), 4.99 (s, 2H), 3.76 (s, 3H).; 13C NMR (100 MHz, DMSO)) δ 158.93 (C), 158.84 (C), 152.06 (C), 140.63 (C), 136.97 (C), 132.42 (CH), 129.57 (C), 129.34 (2CH), 128.93 (C), 128.01 (CH), 124.06 (CH), 122.26 (CH), 113.77 (2CH), 113.05 (CH), 110.65 (CH), 108.45 (CH), 104.89 (CH), 68.84 (CH2), 55.06 (CH3).; IR $_{vmax}$ (cm-1) 3279 (N-H), 1640 (C=O), 1558 (N-H amide), 1551, 1440 (N-C=O), 1248,1179,1011 (C-O).; HR ESMS *m/z* 427.0654 [M-H]+. Calc. for $C_{21}H_{19}N_2O_3Br$ 427.0657.

1-(3-((4-methoxybenzyl)oxy)phenyl)-3-(4-methoxyphenyl)urea (11): Yield 94%; white solid; m.p. 187.9-188.3ºC; 1H NMR (400 MHz, DMSO); δ 8.53 (s, 1H), 8.42 (s, 1H), 7.37 (d, J = 8.7 Hz, 2H), 7.34 (d, J = 9.1 Hz, 2H), 7.22 (t, J = 2.2 Hz, 1H), 7.15 (t, J = 8.1 Hz, 1H), 6.94 (d, J = 8.8 Hz, 2H), 6.93 – 6.90 (m, 1H), 6.86 (d, J = 9.1 Hz, 2H),

6.60 (dd, J = 8.2, 2.5 Hz, 1H), 4.98 (s, 2H), 3.76 (s, 3H), 3.71 (s, 3H).; 13C NMR (100 MHz, DMSO)) δ 158.92 (C), 158.79 (C), 154.47 (C), 152.59 (C), 141.05 (C), 132.60 (C), 129.41 (CH), 129.34 (2CH), 128.98 (C), 120.03 (2CH), 113.96 (2CH), 113.76 (2CH), 110.54 (CH), 107.86 (CH), 104.77 (CH), 68.80 (CH2), 55.14 (CH3), 55.06 (CH3).; IR $_{vmax}$ (cm-1) 3289 (N-H), 1637 (C=O), 1597 (N-H amide), 1508 (N-C=O), 1241,1182,1031 (C-O).; HR ESMS *m/z* 401.1471 [M-Na]+. Calc. for $C_{22}H_{22}N_2O_3$ 401.1477.

1-(3-((4-methoxybenzyl)oxy)phenyl)-3-(3-methoxyphenyl)urea (12): Yield 80%; white solid; m.p. 137.5-168.9ºC; 1H NMR (400 MHz, DMSO); δ 8.64 (s, 1H), 8.61 (s, 1H), 7.38 (d, J = 8.7 Hz, 2H), 7.22 (t, J = 2.2 Hz, 1H), 7.20 – 7.13 (m, 3H), 6.98 – 6.89 (m, 4H), 6.62 (dd, J = 8.2, 2.5 Hz, 1H), 6.55 (dd, J = 8.3, 2.5 Hz, 1H), 4.99 (s, 2H), 3.76 (s, 3H), 3.73 (s, 3H).; 13C NMR (100 MHz, DMSO)) δ 159.67 (C), 158.94 (C), 158.82 (C), 152.34 (C), 140.82 (C), 140.80 (C), 129.50 (CH), 129.48 (CH), 129.37 (2CH), 128.97 (C), 113.78 (2CH), 110.71 (CH), 110.53 (CH), 108.09 (CH), 107.24 (CH), 104.96 (CH), 104.01 (CH), 68.85 (CH2), 55.07 (CH3), 54.90 (CH3).; IR $_{vmax}$ (cm-1) 3289 (N-H), 1624 (C=O), 1561 (N-H amide), 1515 (N-C=O), 1258,1169,1028 (C-O).; HR ESMS *m/z* 379.1659 [M-H]+. Calc. for $C_{22}H_{22}N_2O_3$ 379.1658.

1-(3-((4-methoxybenzyl)oxy)phenyl)-3-(2-methoxyphenyl)urea (13): Yield 44%; white solid; m.p. 160.0-161.6ºC; 1H NMR (400 MHz, DMSO); δ 9.28 (s, 1H), 8.20 (s, 1H), 8.12 (dd, J = 7.9, 1.8 Hz, 1H), 7.38 (d, J = 8.7 Hz, 2H), 7.25 (t, J = 2.2 Hz, 1H), 7.16 (t, J = 8.1 Hz, 1H), 7.01 (dd, J = 8.0, 1.5 Hz, 1H), 6.98 – 6.91 (m, 4H), 6.91 – 6.86 (m, 1H), 6.61 (dd, J = 8.2, 2.5 Hz, 1H), 4.99 (s, 2H), 3.88 (s, 3H), 3.76 (s, 3H).; 13C NMR (100 MHz, DMSO)) δ 158.93 (C), 158.83 (C), 152.27 (C), 147.63 (C), 141.00 (C), 129.49 (CH), 129.34 (2CH), 128.97 (C), 128.58 (C), 121.78 (CH), 120.50 (CH), 118.30 (CH), 113.77 (CH), 110.71 (CH), 110.39 (CH), 108.02 (CH), 104.62 (CH), 68.81 (CH2), 55.74 (CH3), 55.06 (CH3).; IR $_{vmax}$ (cm-1) 3329 (N-H), 1650 (C=O), 1541 (N-H amide), 1518, 1466 (N-C=O), 1245,1156,1031 (C-O).; HR ESMS *m/z* 379.1656 [M-H]+. Calc. for $C_{22}H_{22}N_2O_3$ 379.1658.

Phenyl (3-((4-methoxybenzyl)oxy)phenyl)carbamate (14): Yield 78%; white solid; m.p.170.3ºC; 1H NMR (400 MHz, $CDCl_3$); δ 7.42 (t, J = 7.8 Hz, 2H,CH-23,24), 7.37 (d, J = 8.4 Hz, 2H, CH-1,5), 7.31 (s, 1H,NH), 7.27 (d, J = 6.3 Hz, 2H, CH-22,26), 7.22 (t, J

= 7.6 Hz, 2H, CH-24,16), 6.98-6.87 (m, 4H, CH-2,4,13,14), 6.74 (dd, J = 8.3, 2.4 Hz, 1H, CH-12), 5.00 (s, 2H, O-CH2-Ar), 3.83 (s, 3H, OCH3).; 13C NMR (100 MHz, $CDCl_3$) δ 159.62(C), 159.48(C), 151.51(C), 150.54(C), 138.58(C), 129.85(CH), 129.41(2CH), 129.22(2CH), 128.87(C), 125.73(CH), 121.63(2CH), 114.01(2CH), 111.10(CH), 110.86(CH), 105.34(CH), 69.83(CH2), 55.29(CH3); IR $_{vmax}$ (cm-1) 3240 (N-H), 1703 (C=O), 1542 (N-H amide), 1445 (N-C=O), 1235,1205,1008 (C-O); HR ESMS *m/z* 372.1211 [M-Na]+. Calc. for $C_{21}H_{19}N_1O_4$ 372.1212.

Graphical NMR spectra

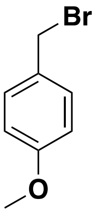

I.2.

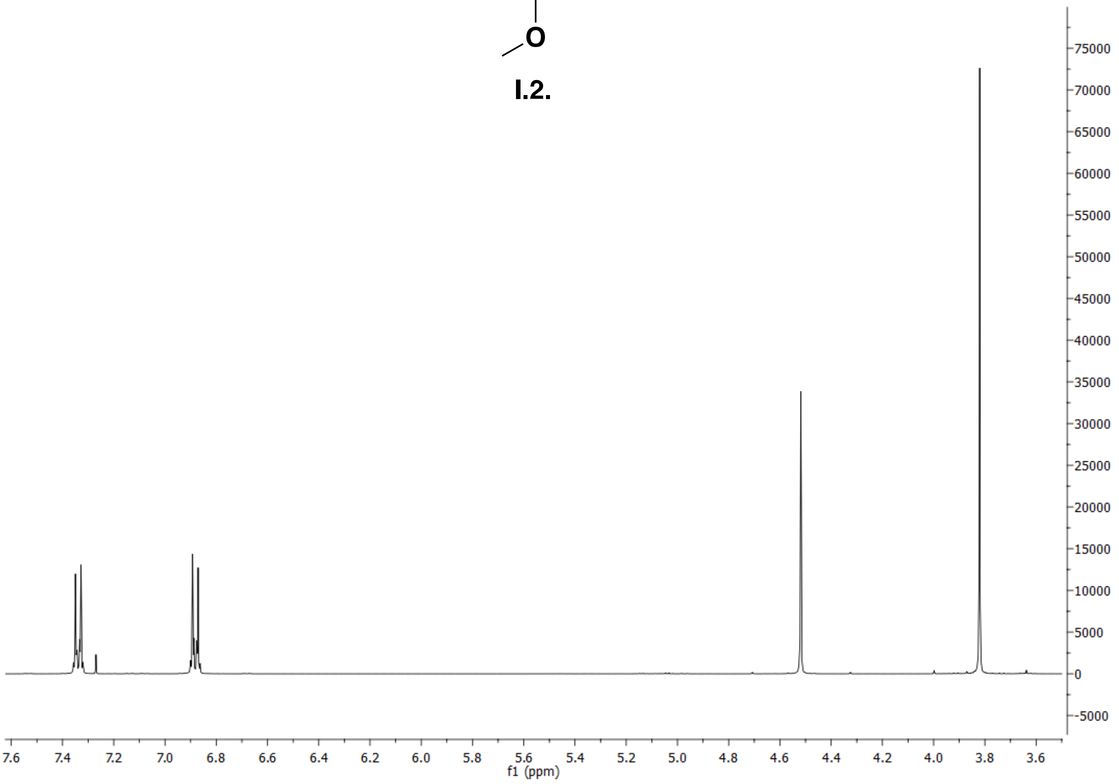

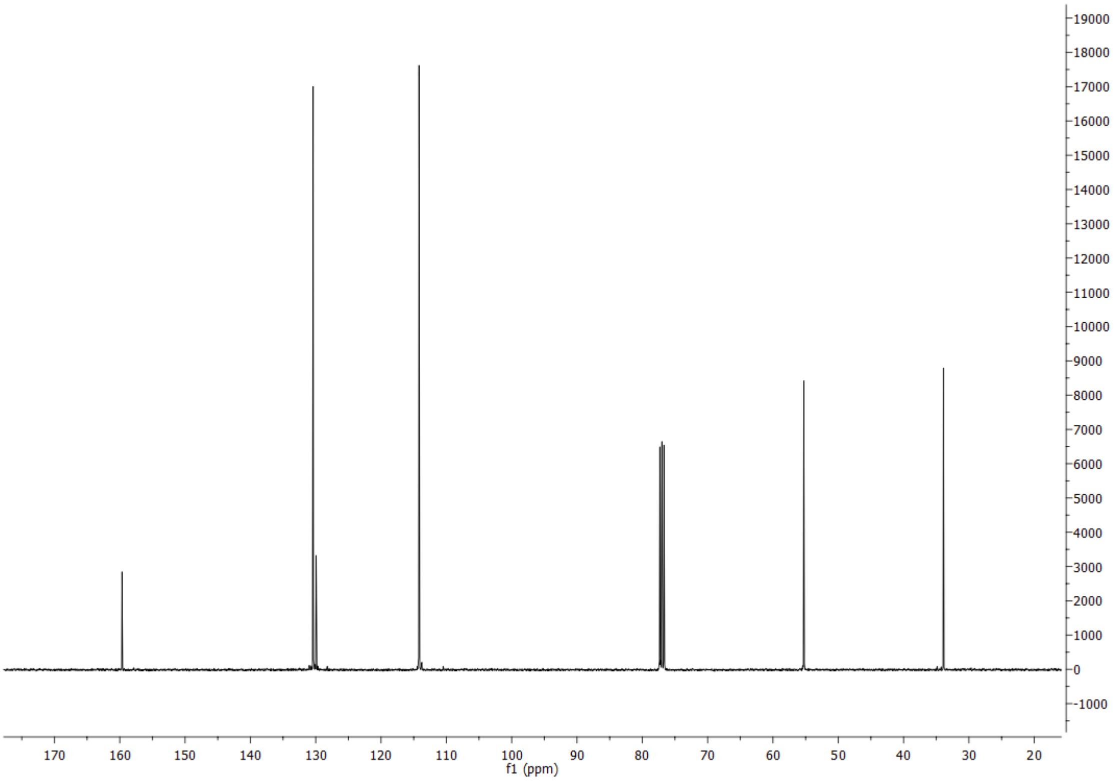

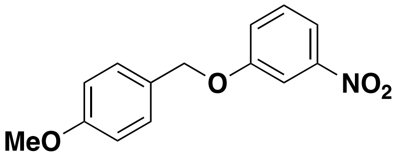

I.4.

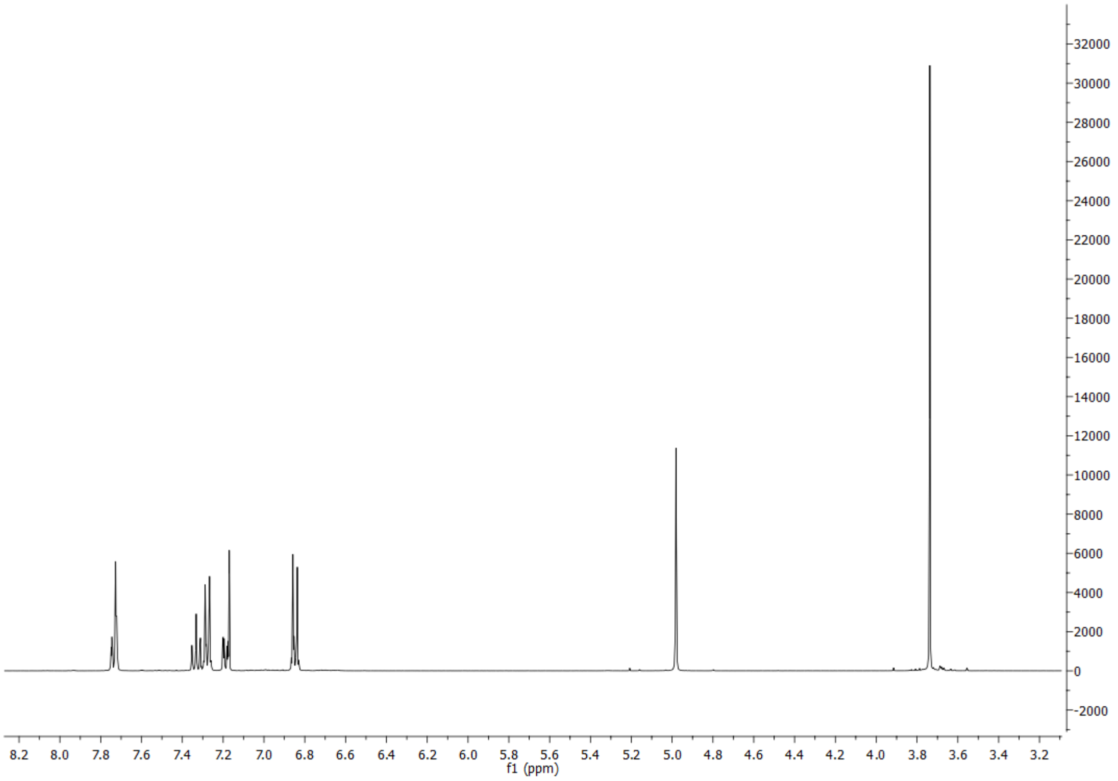

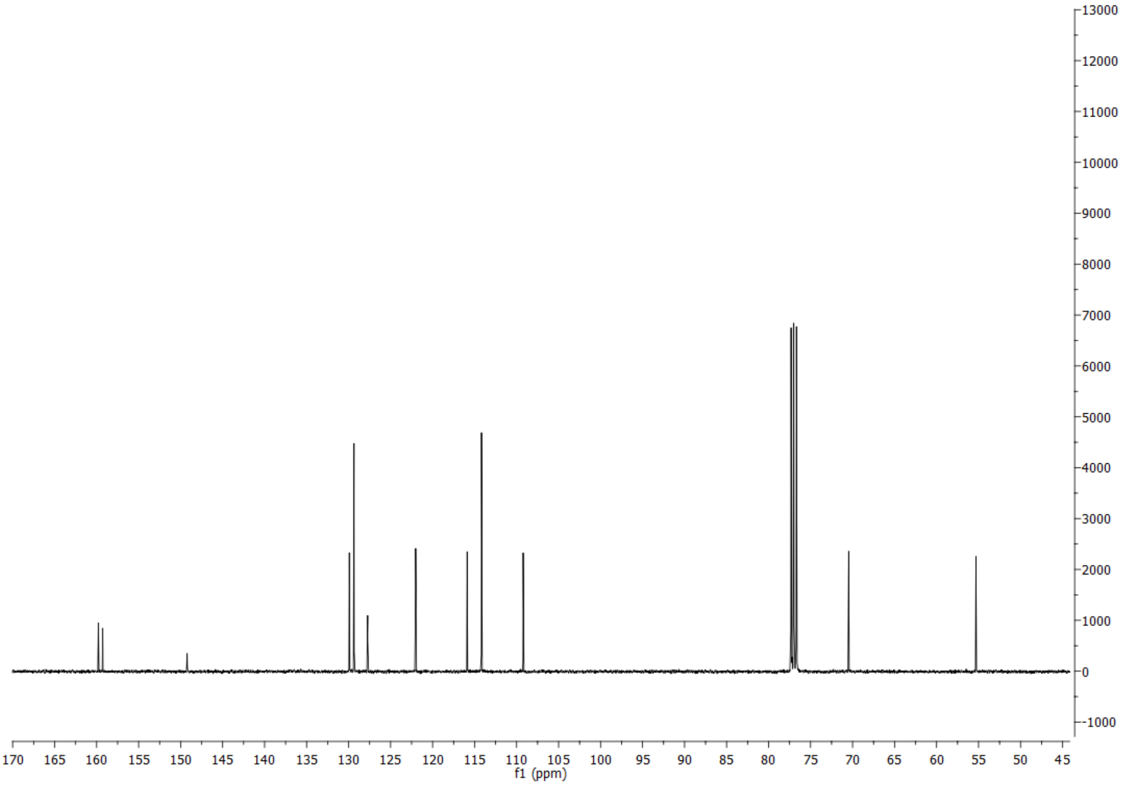

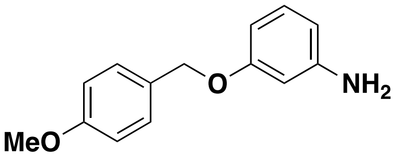

I.5.

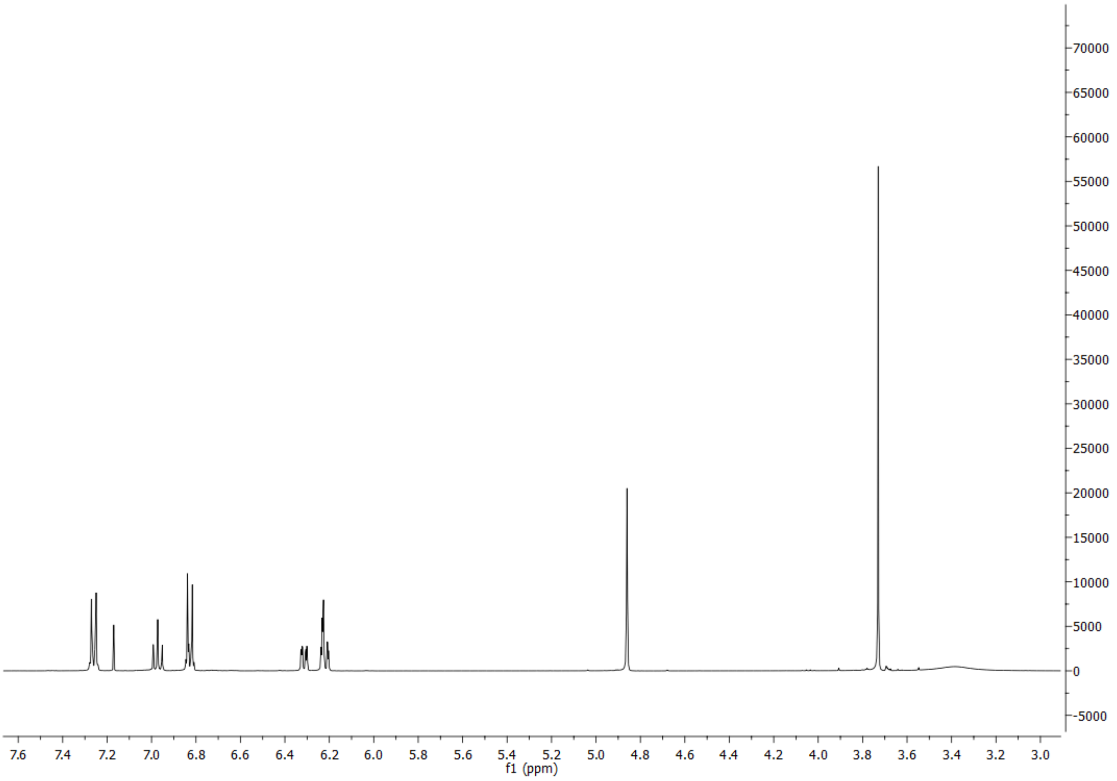

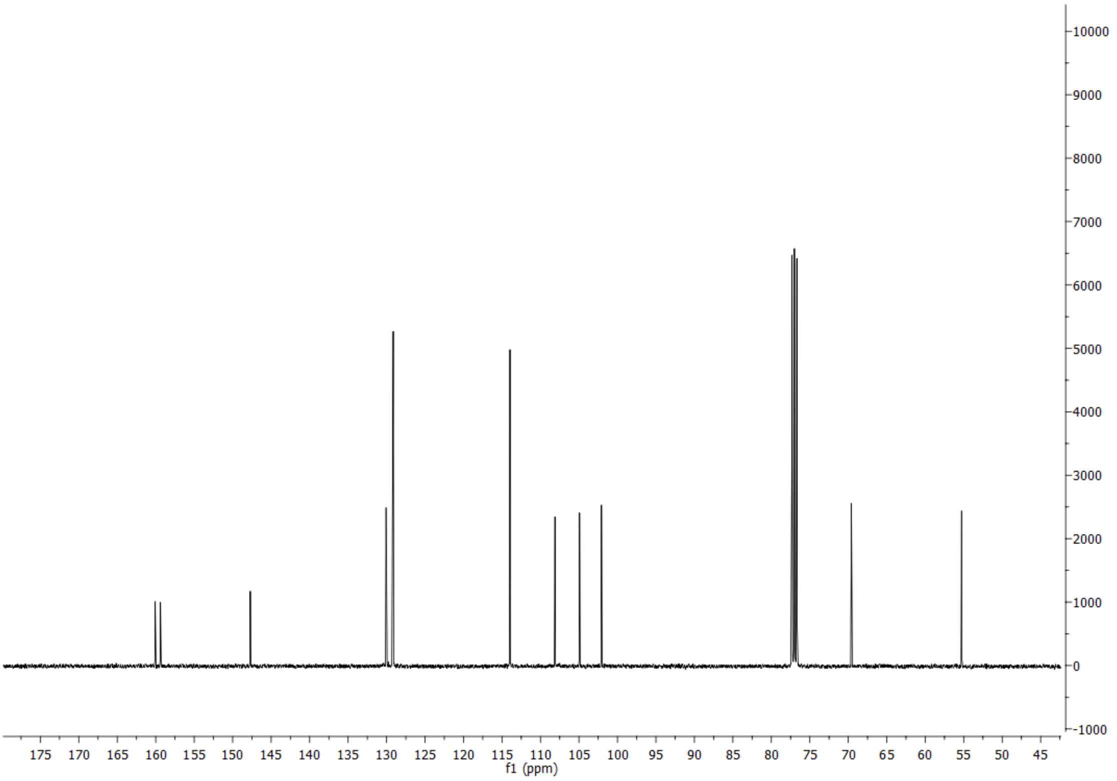

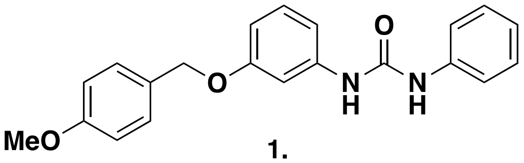

1.

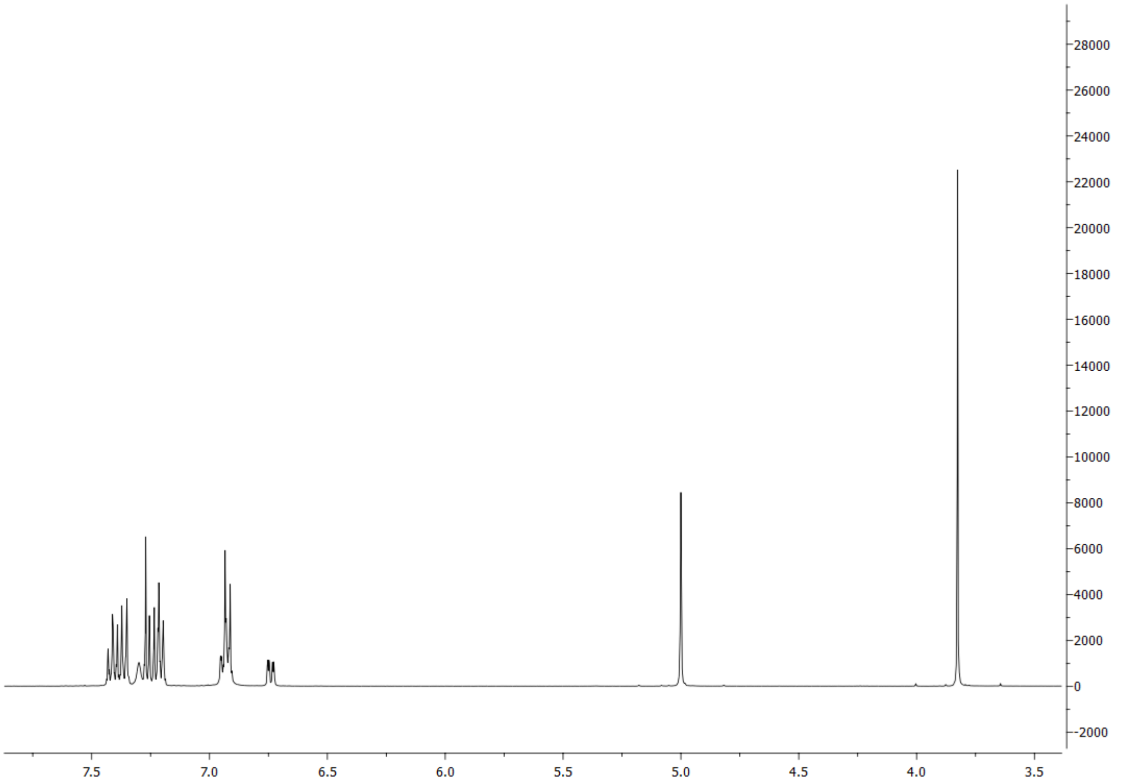

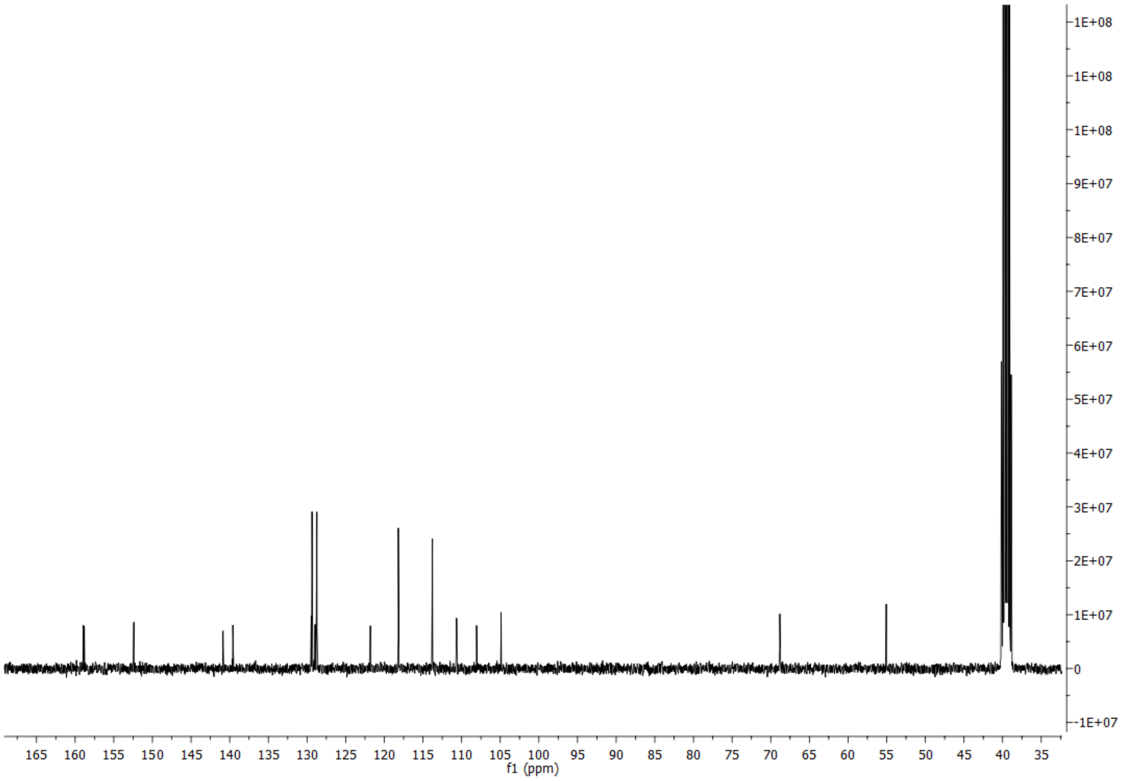

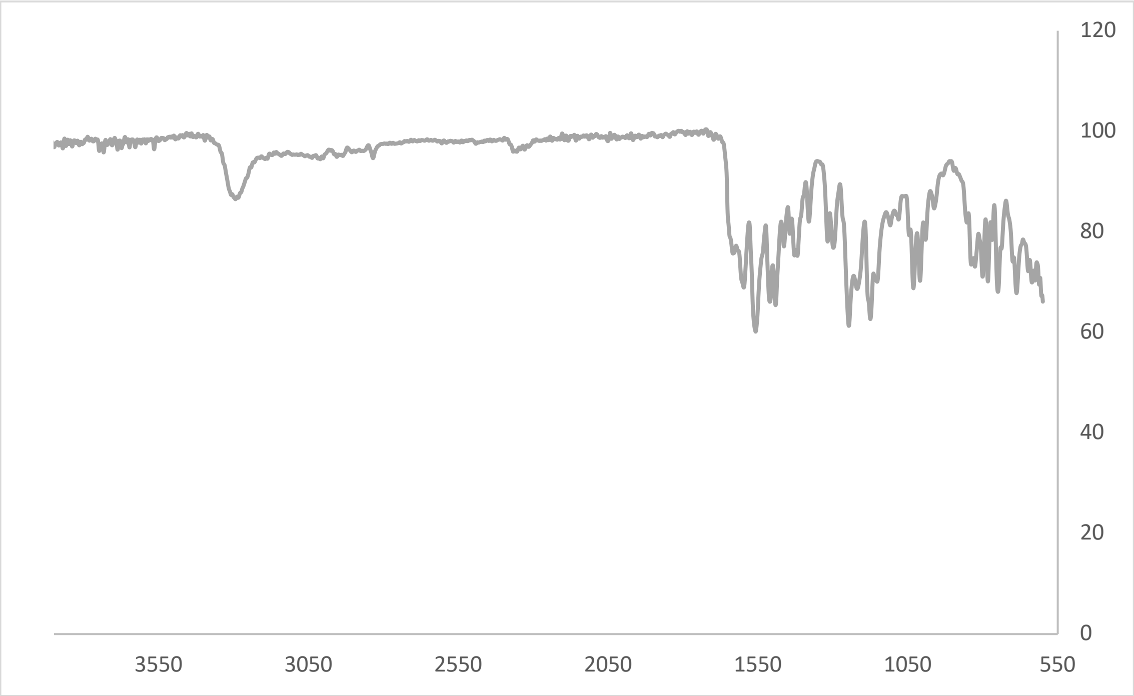
120
100
80
60
40
20
0
3550
3050
2550
2050
1550
1050
550

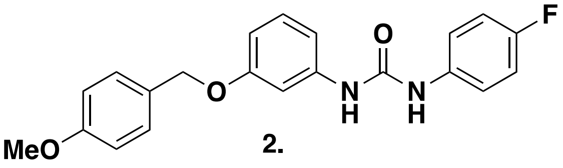

2.

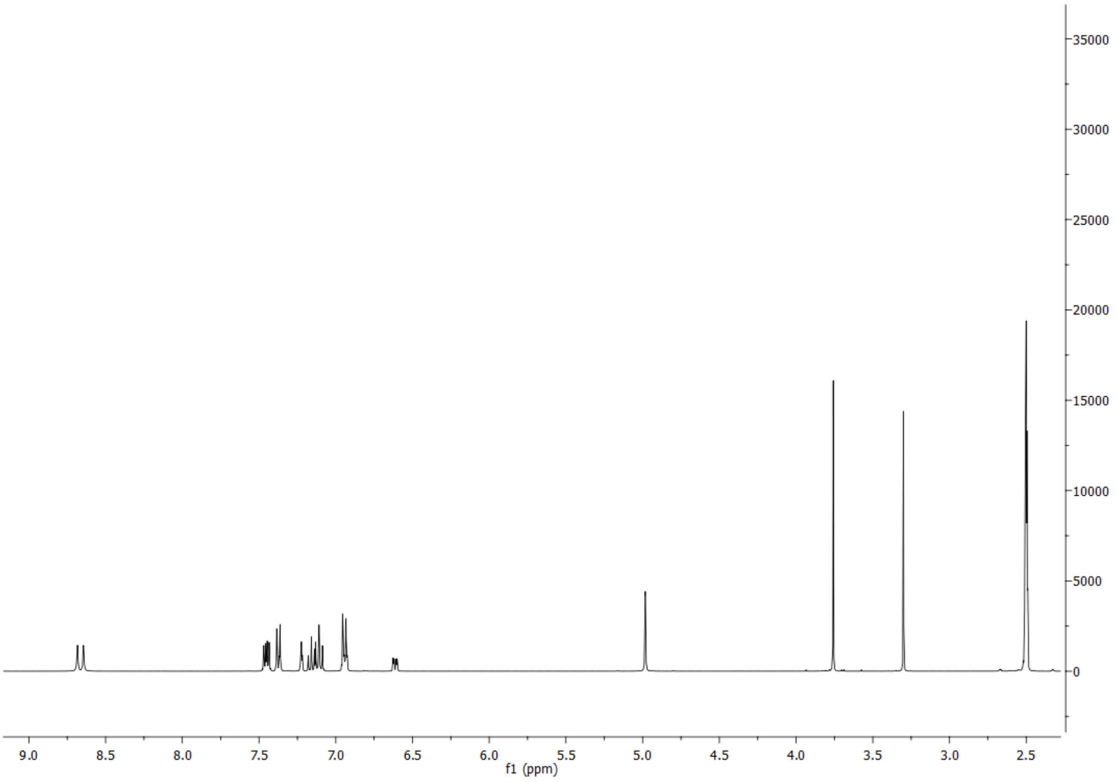

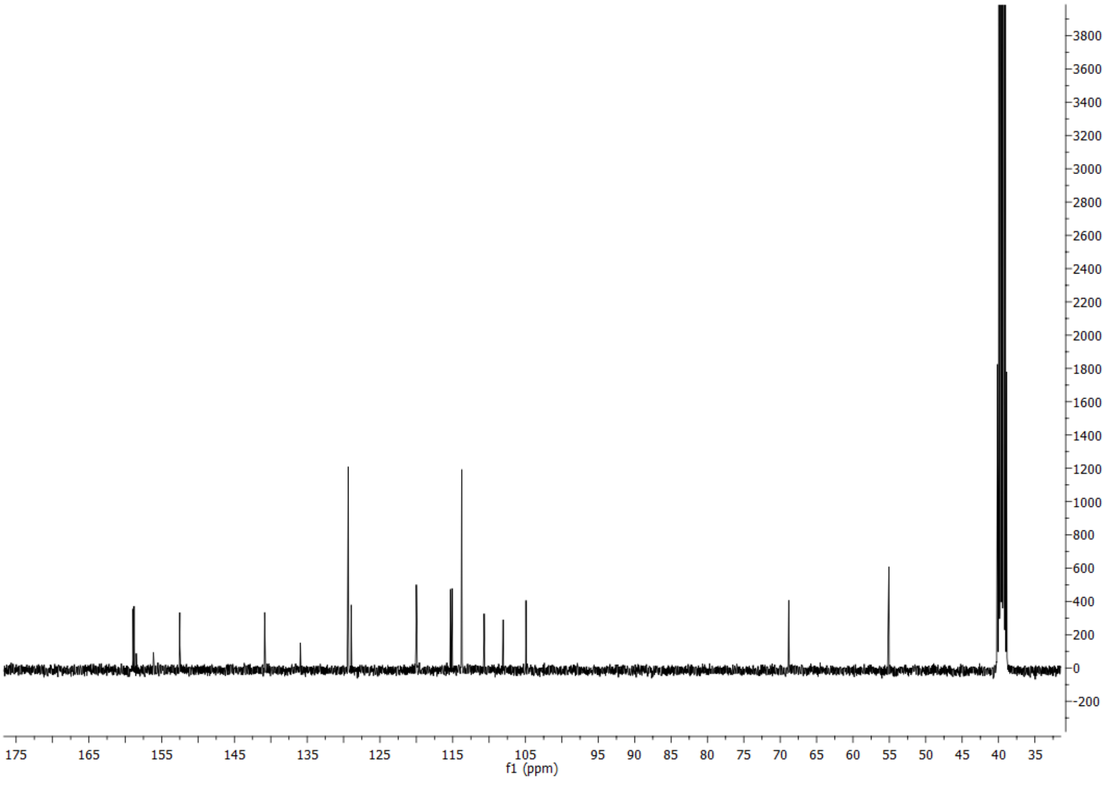

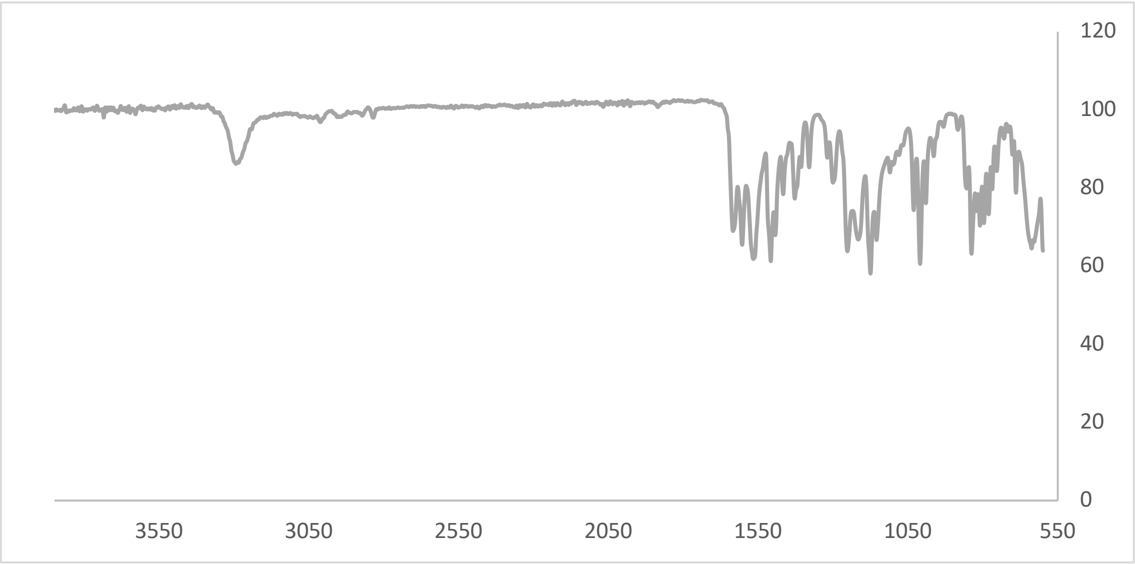
120
100
80
60
40
20
0
3550
3050
2550
2050
1550
1050
550

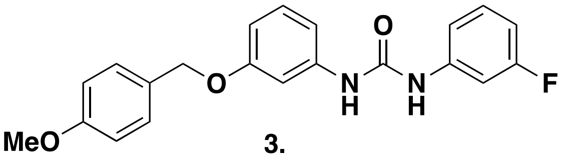

3.

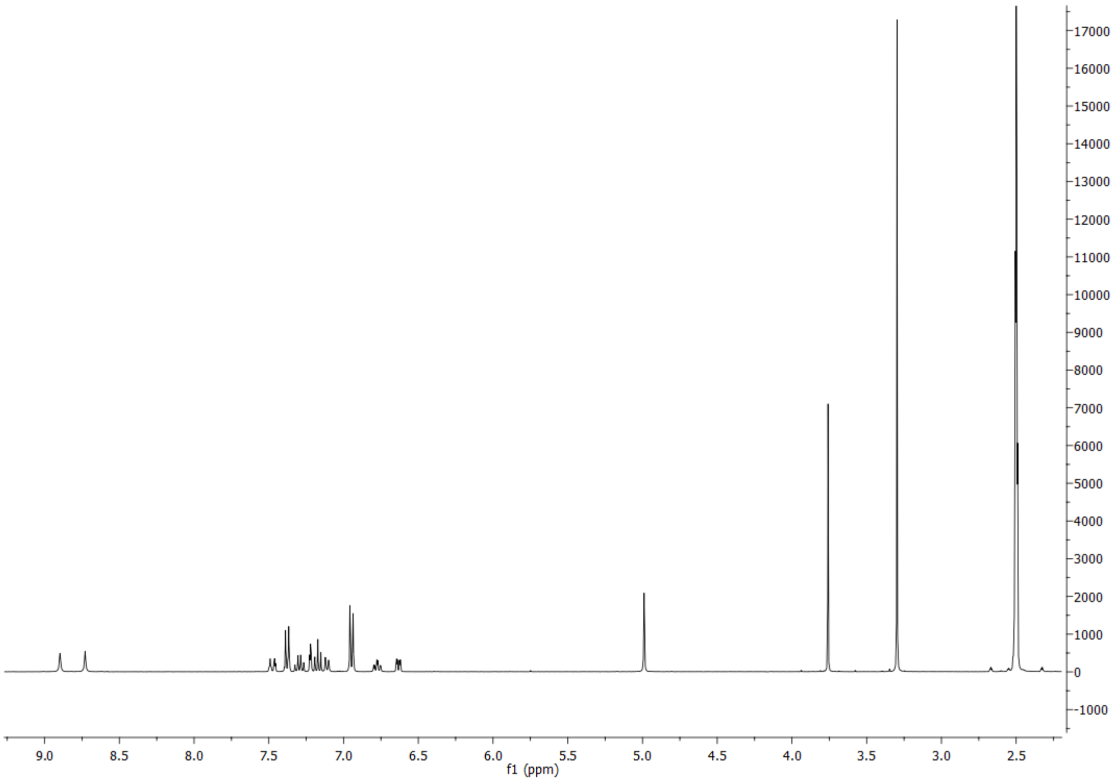

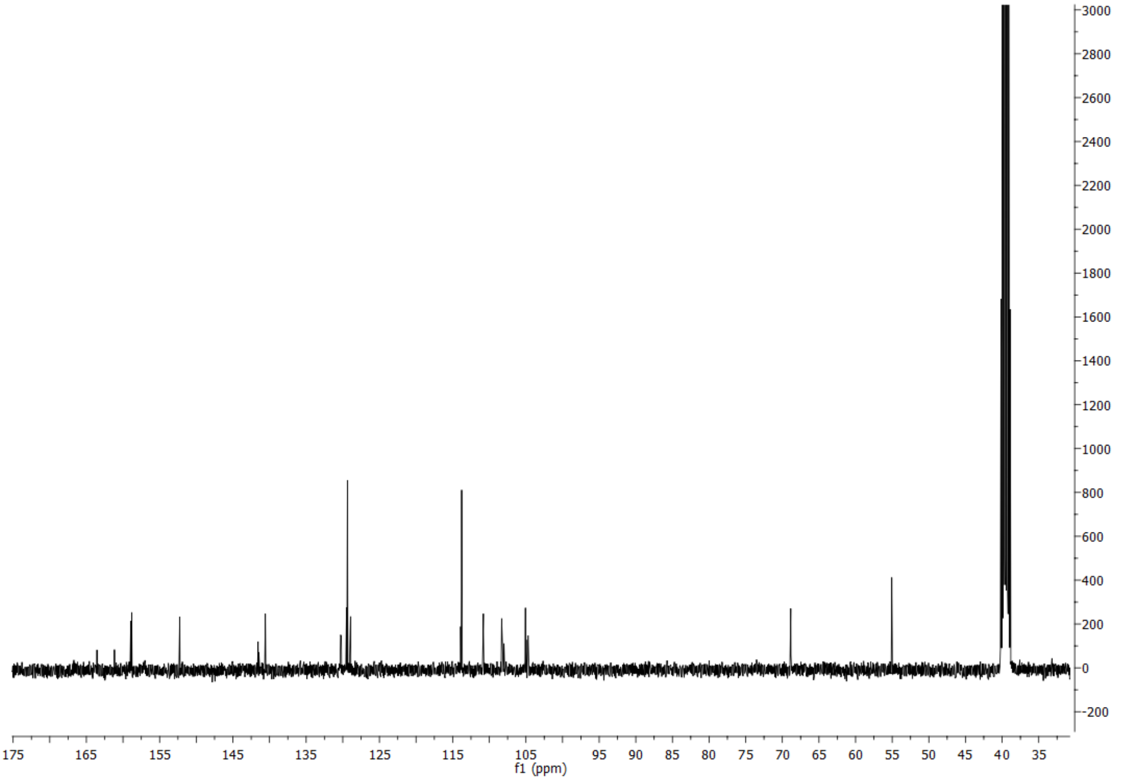

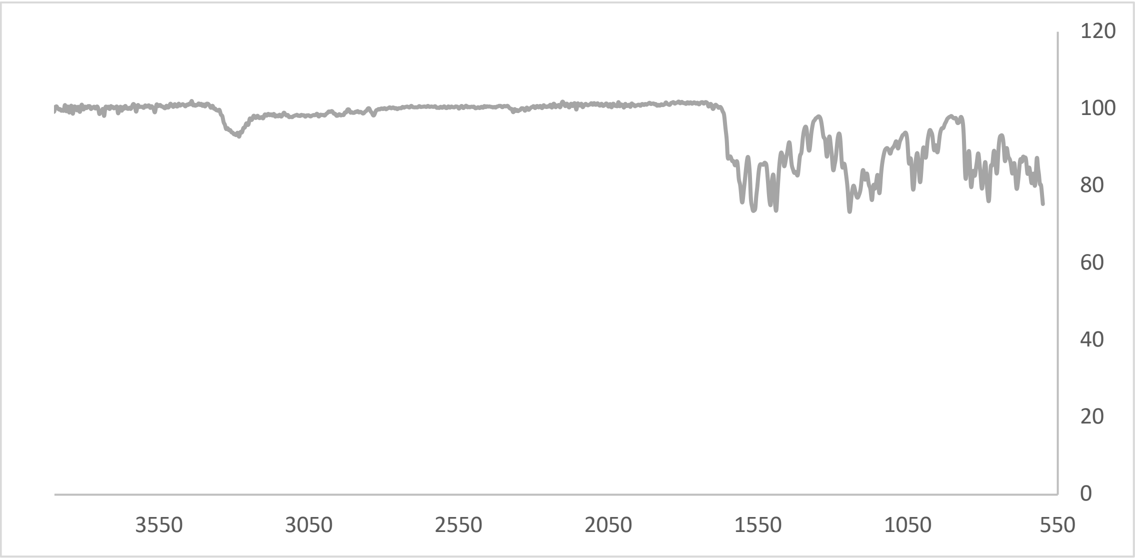
120
100
80
60
40
20
0
3550
3050
2550
2050
1550
1050
550

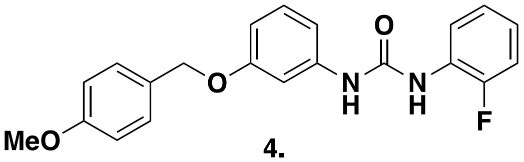
MeO
O
O
N
H
N
H
F

4.

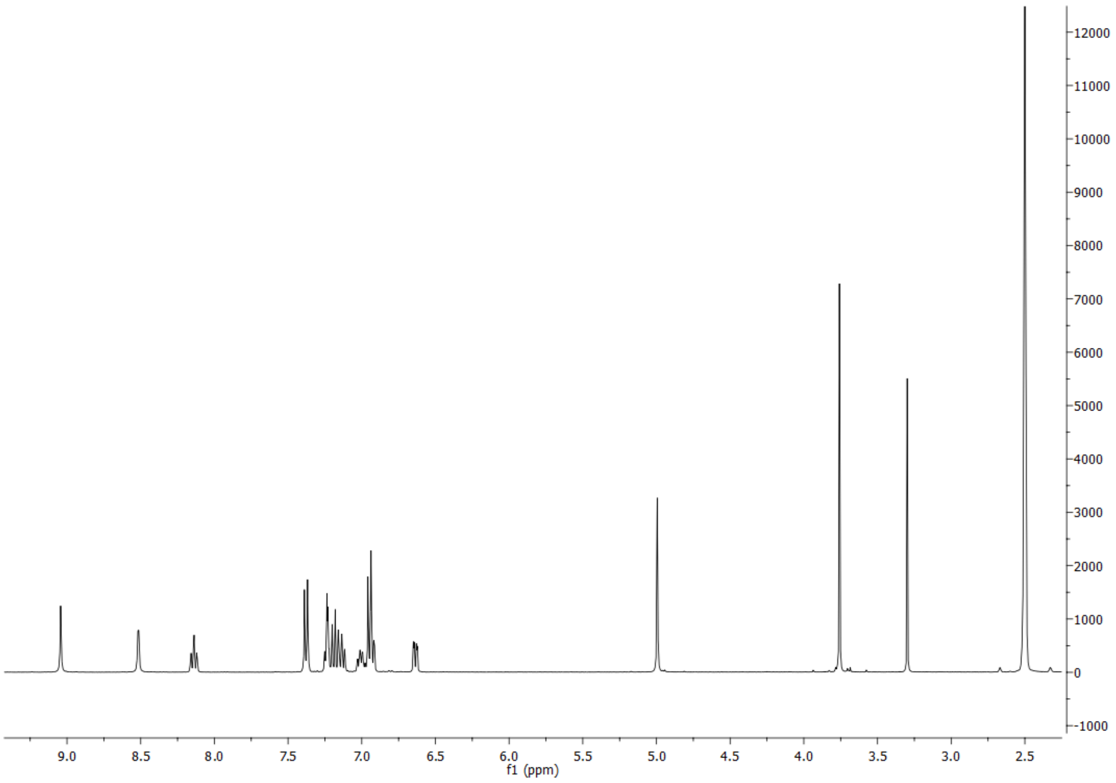
9.0 8.5 8.0 7.5 7.0 6.5 6.0 5.5 5.0 4.5 4.0 3.5 3.0 2.5
f1 (ppm)
12000 11000 10000 9000 8000 7000 6000 5000 4000 3000 2000 1000 0 -1000

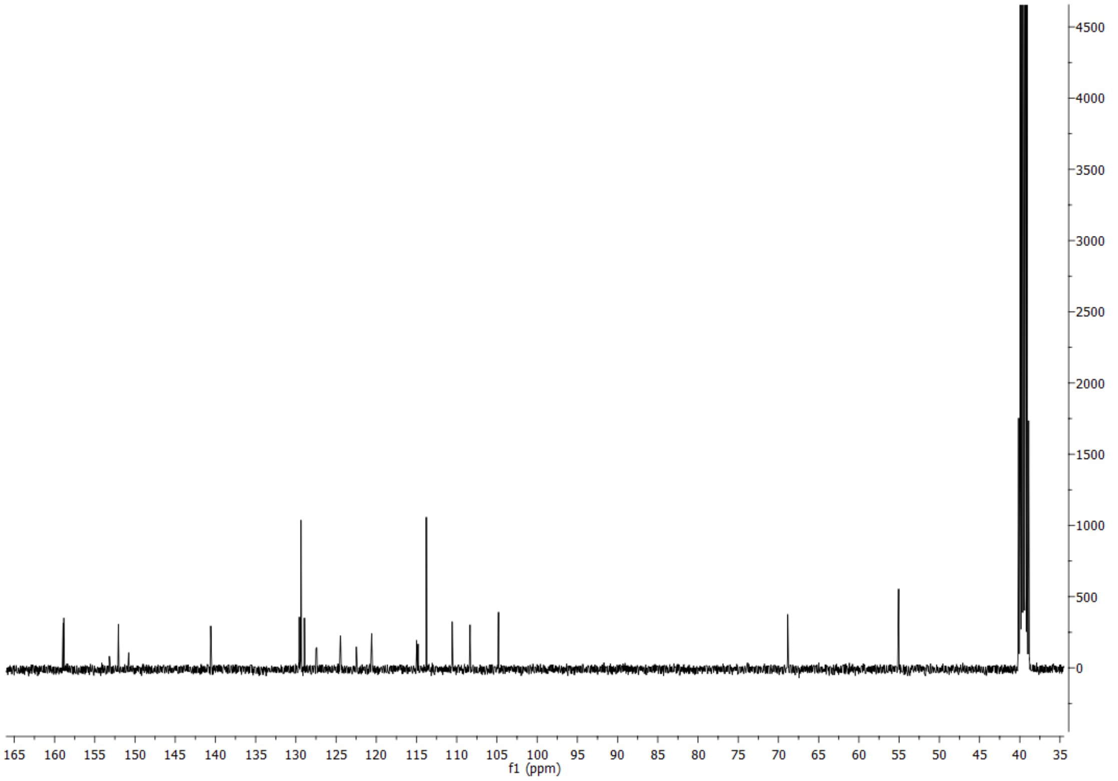
165 160 155 150 145 140 135 130 125 120 115 110 105 100 95 90 85 80 75 70 65 60 55 50 45 40 35
f1 (ppm)
4500 4000 3500 3000 2500 2000 1500 1000 500 0

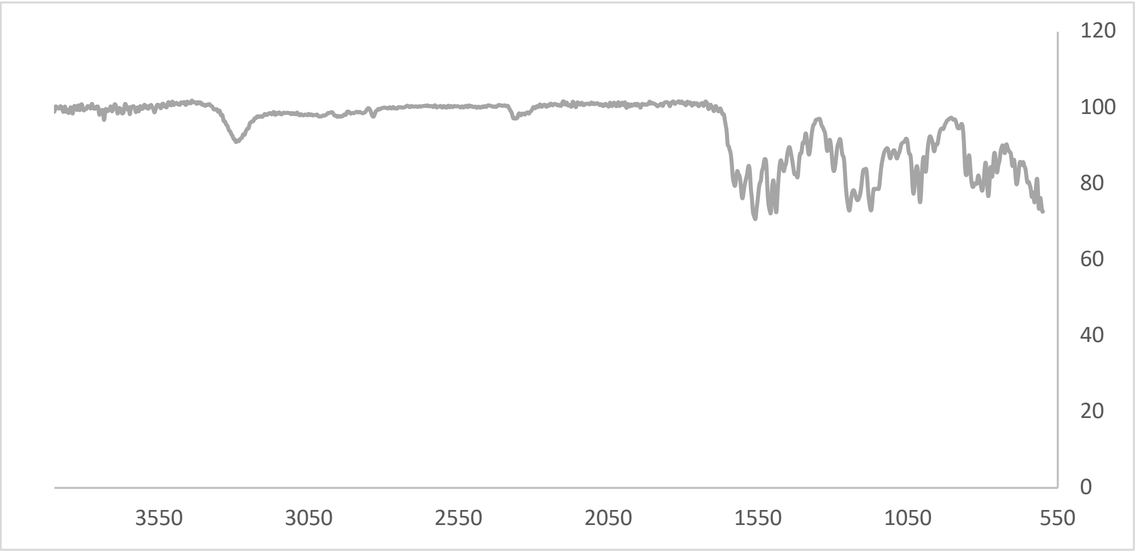
120
100
80
60
40
20
0
3550
3050
2550
2050
1550
1050
550

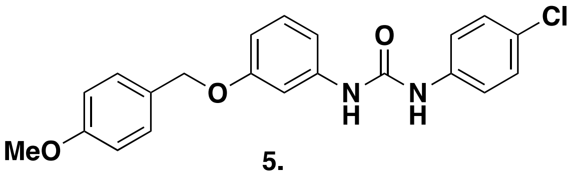
MeO
O
N
H
O
N
H
Cl

5.

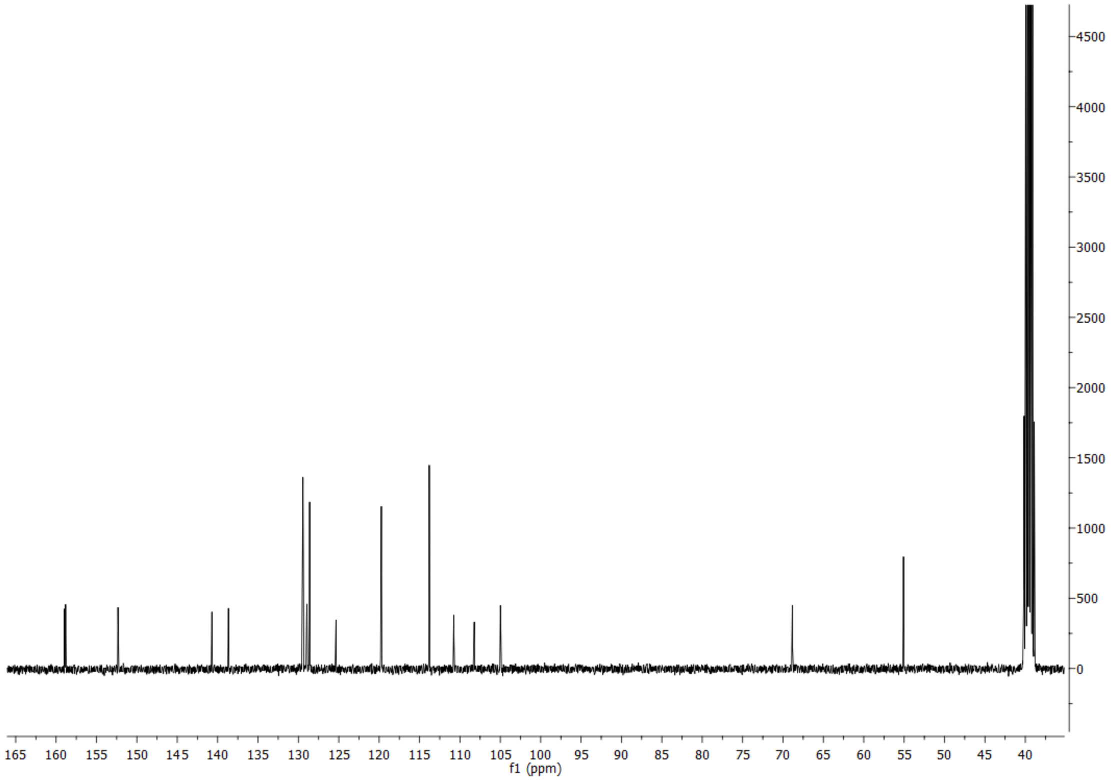
165 160 155 150 145 140 135 130 125 120 115 110 105 100 95 90 85 80 75 70 65 60 55 50 45 40
f1 (ppm)
4500 4000 3500 3000 2500 2000 1500 1000 500 0

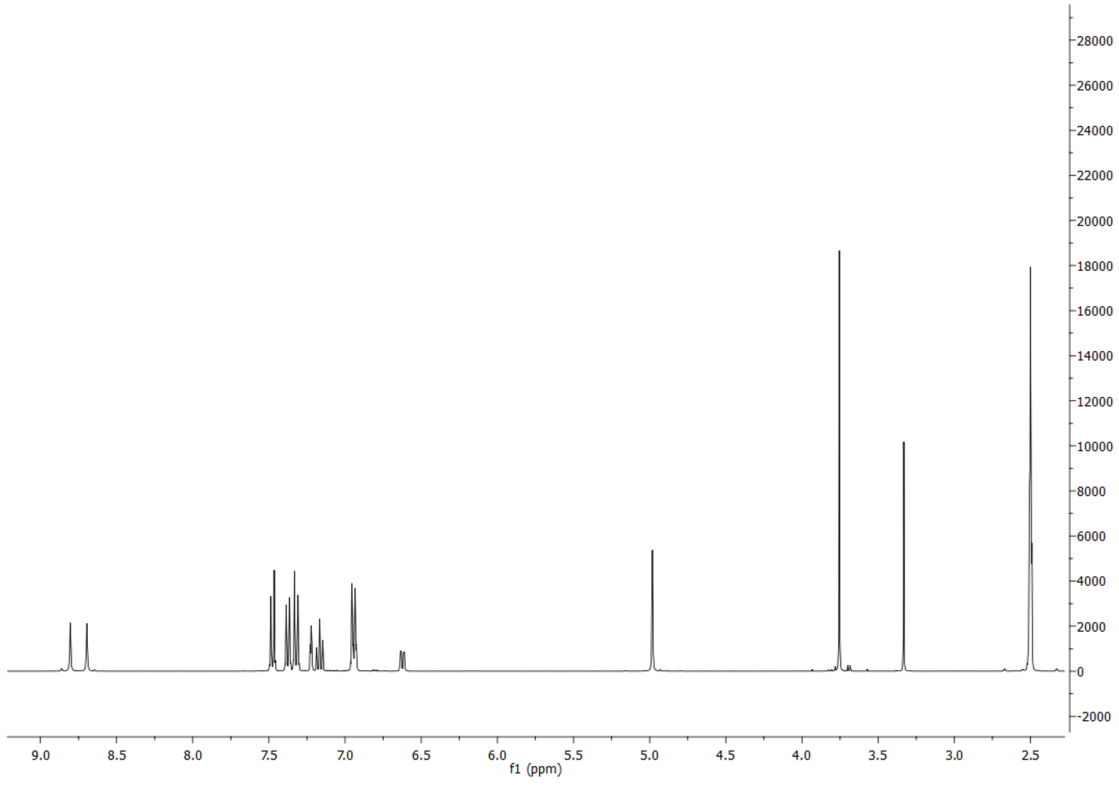
9.0 8.5 8.0 7.5 7.0 6.5 6.0 5.5 5.0 4.5 4.0 3.5 3.0 2.5
f1 (ppm)
28000 26000 24000 22000 20000 18000 16000 14000 12000 10000 8000 6000 4000 2000 0 -2000

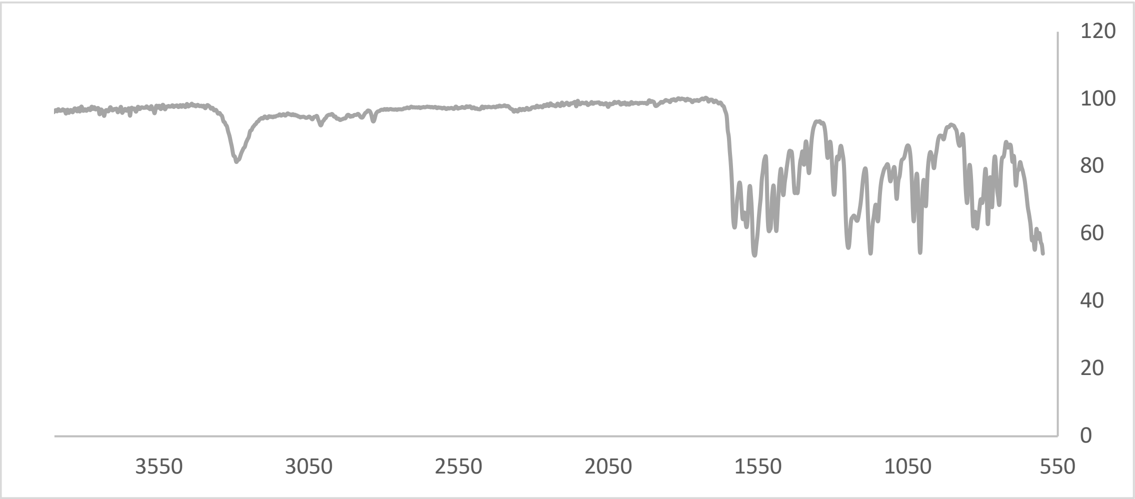
120
100
80
60
40
20
0
3550
3050
2550
2050
1550
1050
550

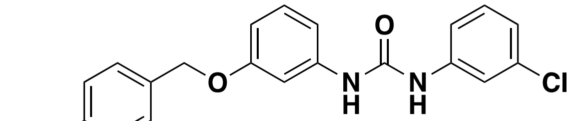

6.

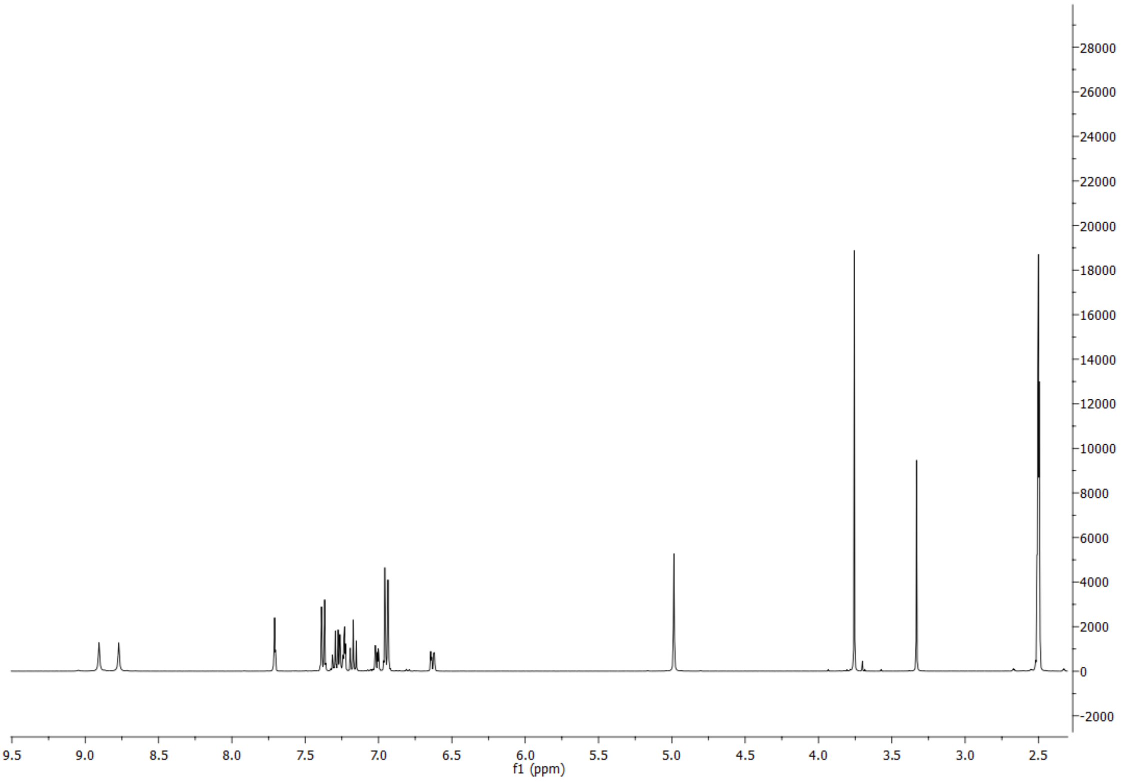

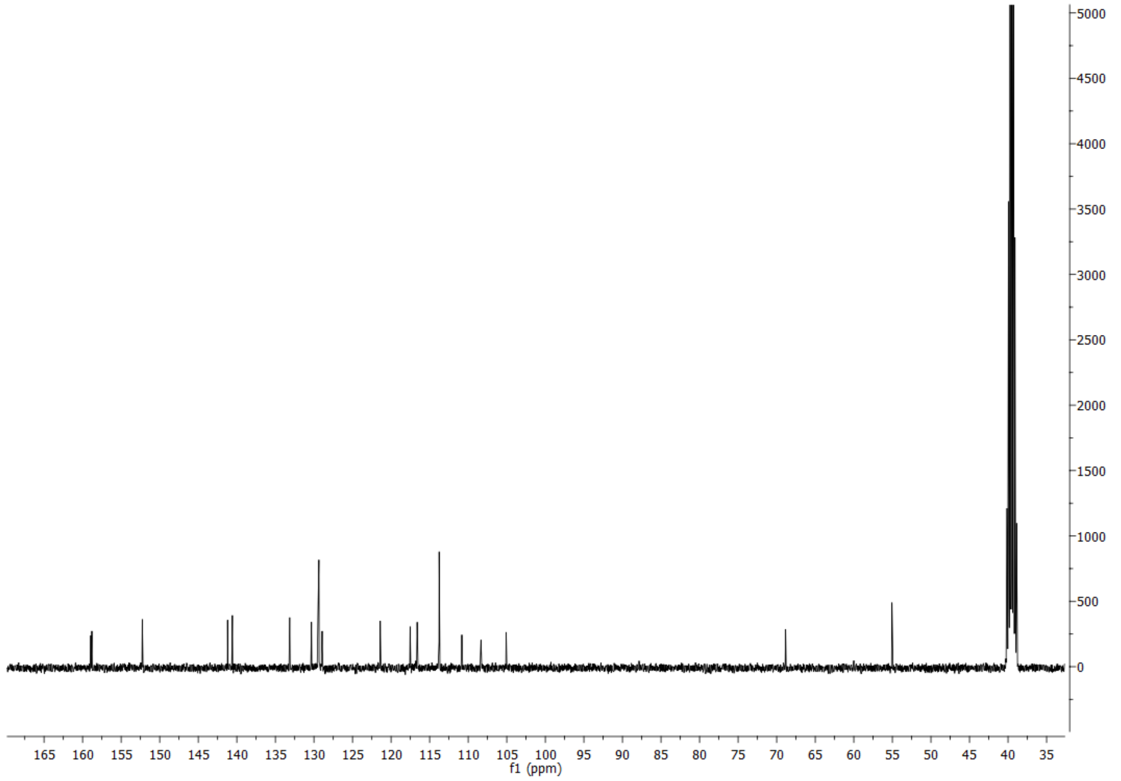

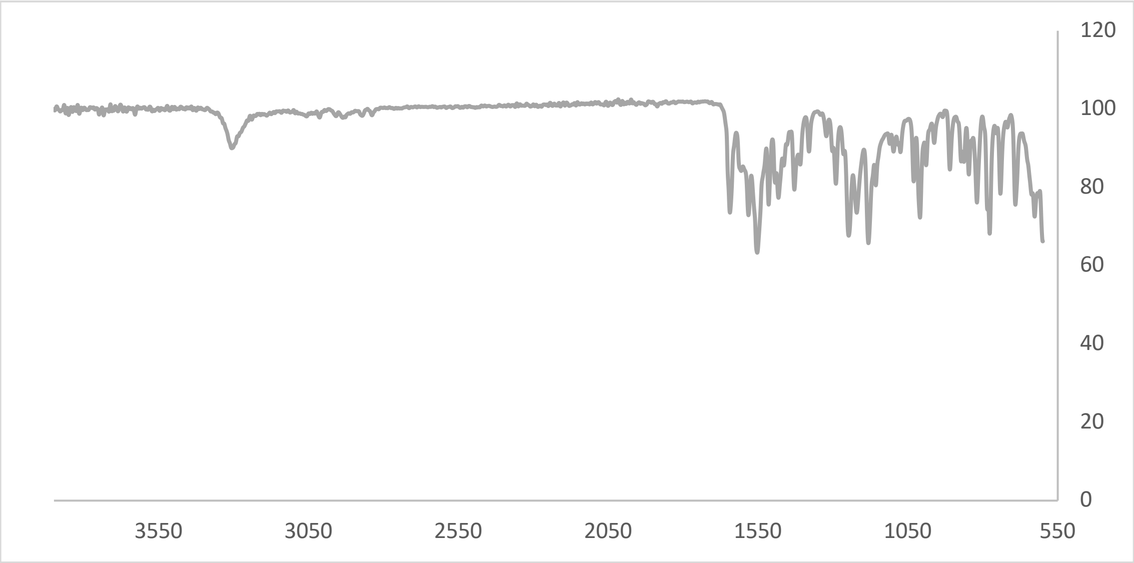
120
100
80
60
40
20
0
3550
3050
2550
2050
1550
1050
550

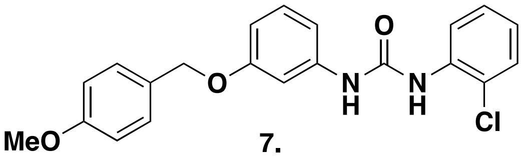

7.

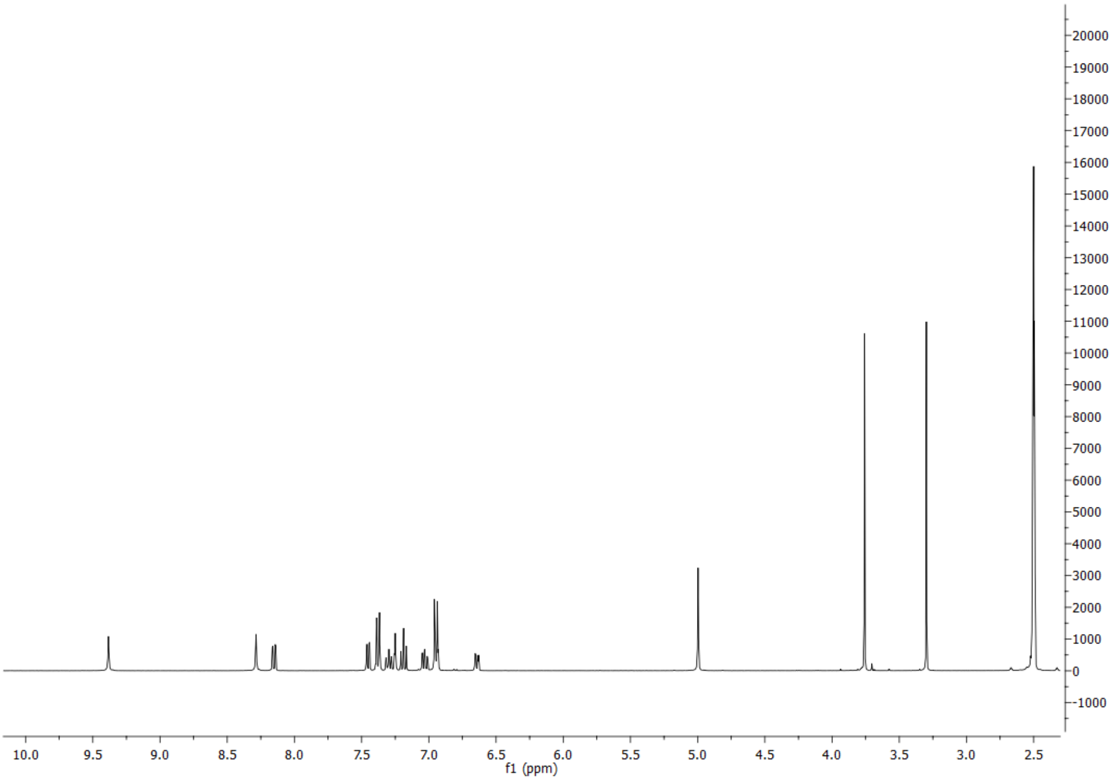

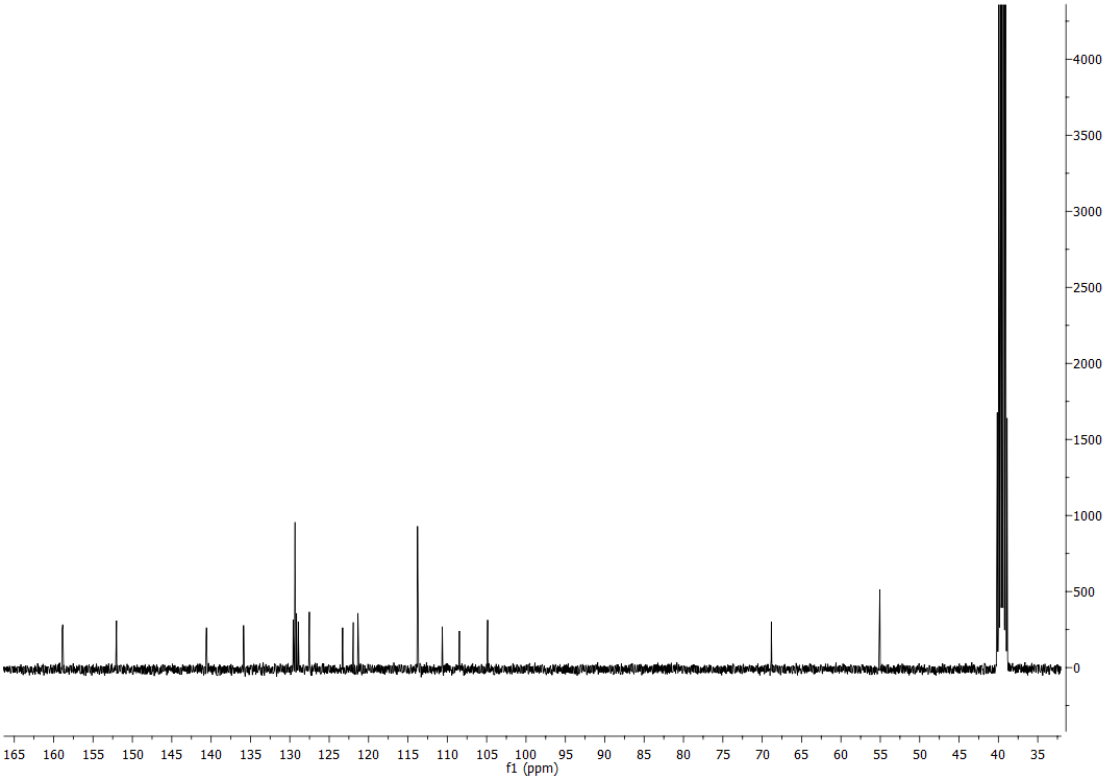

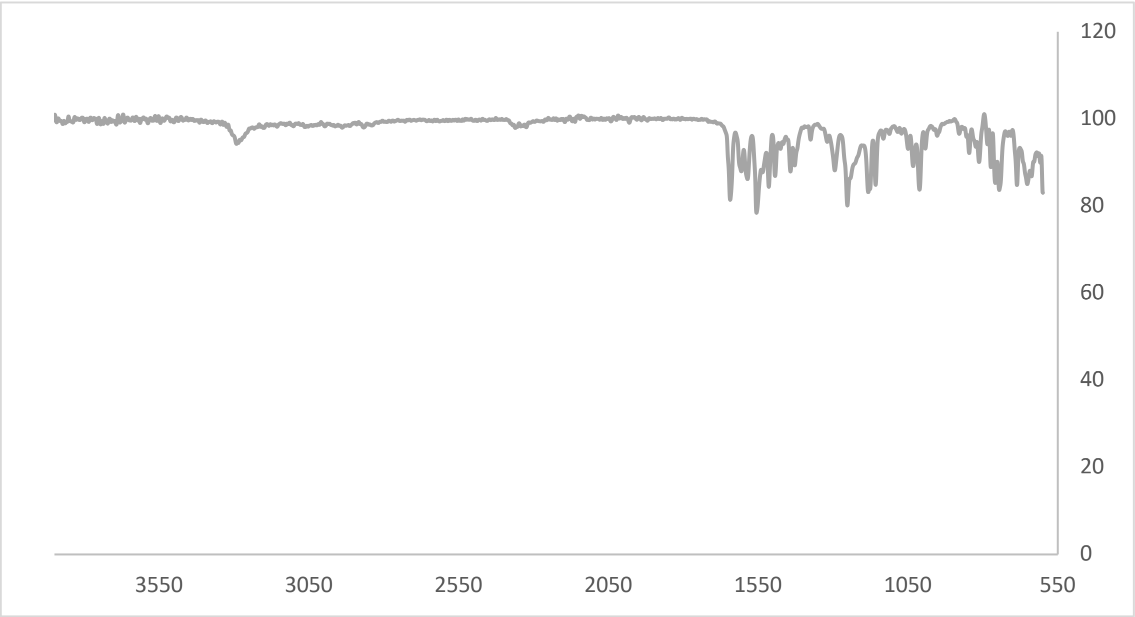
120
100
80
60
40
20
0
3550
3050
2550
2050
1550
1050
550

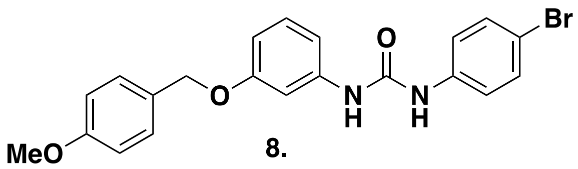

8.

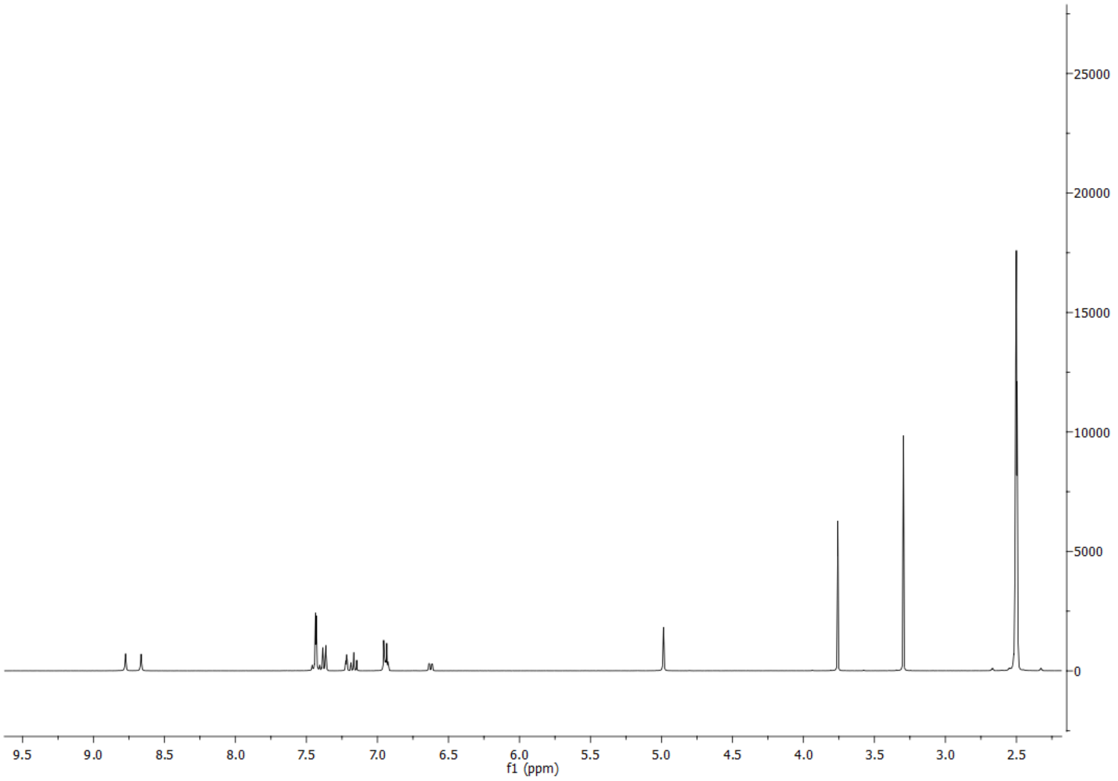

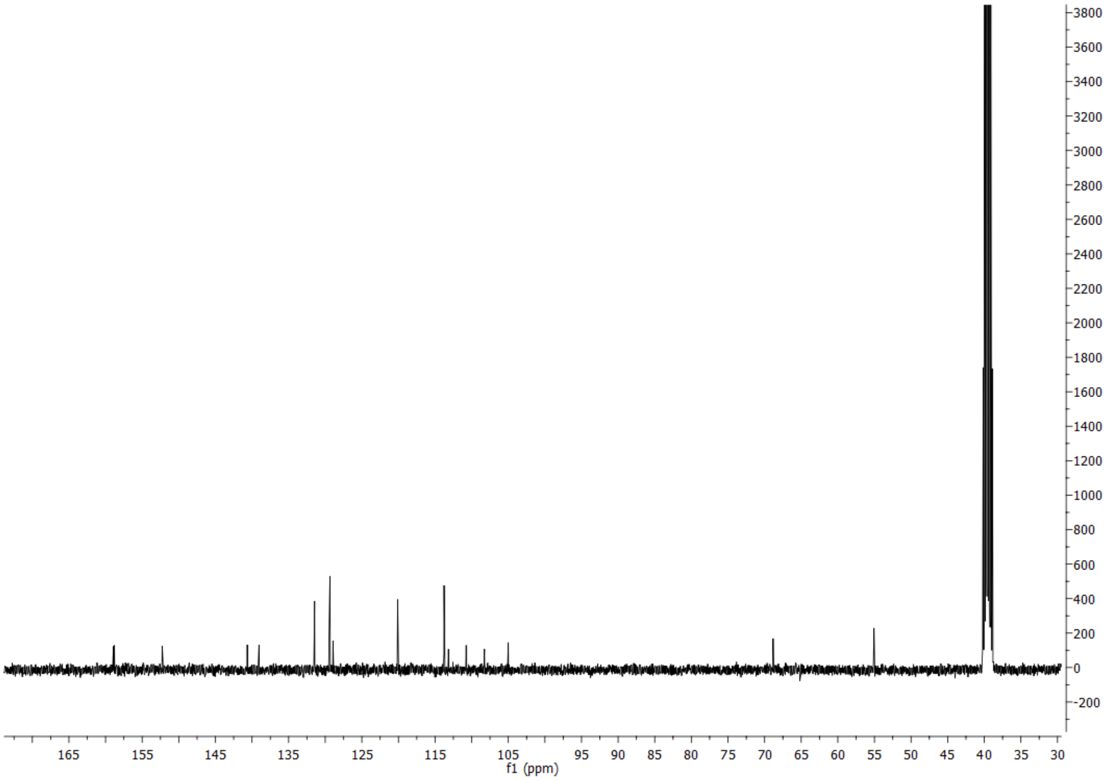

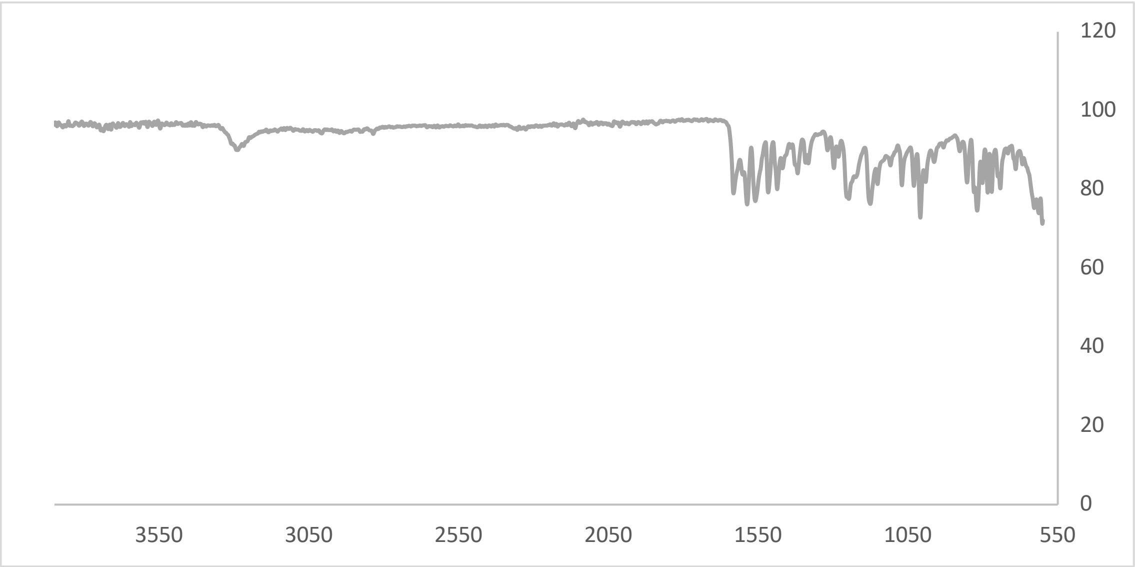
120
100
80
60
40
20
0
3550
3050
2550
2050
1550
1050
550

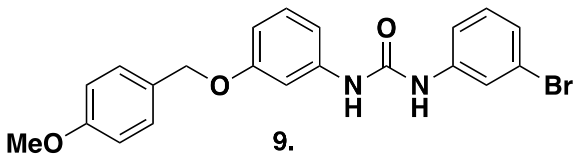

9.

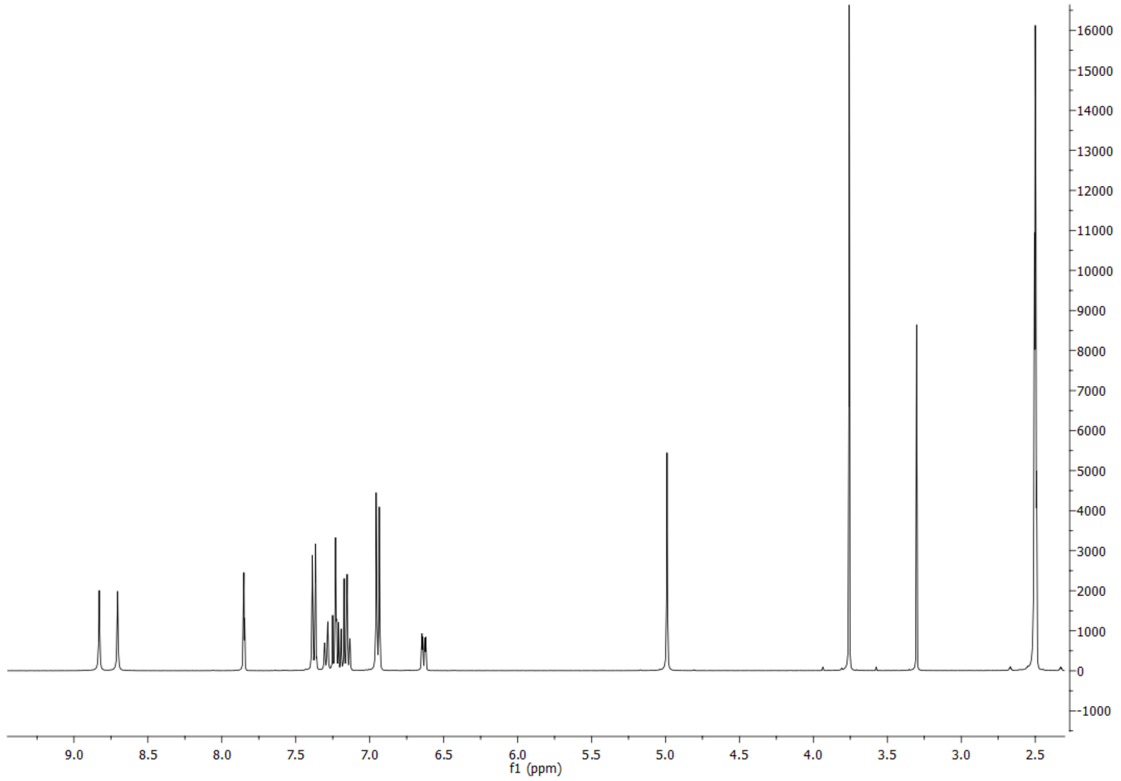

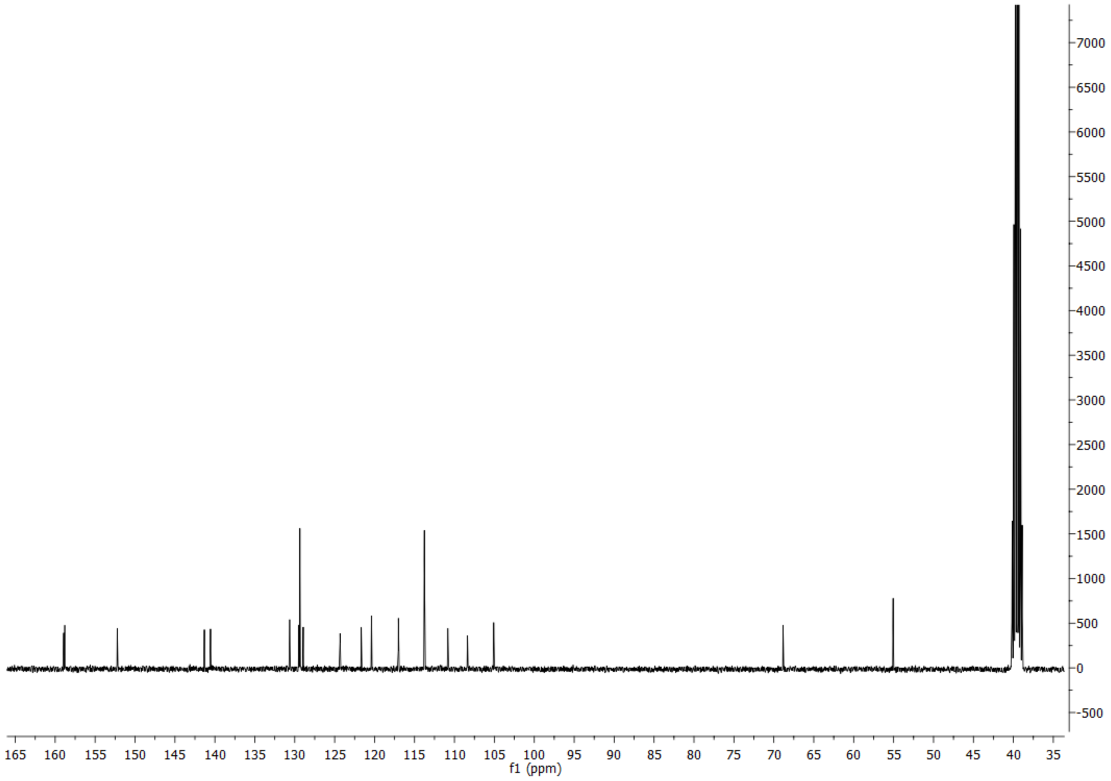

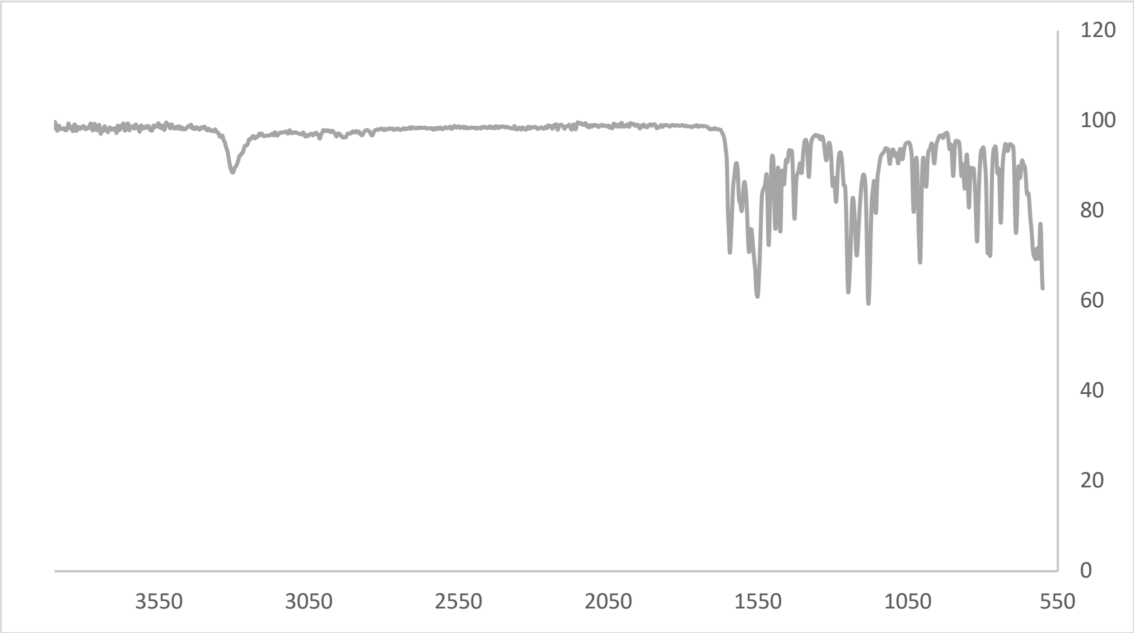

120
100
80
60
40
20
0
3550
3050
2550
2050
1550
1050
550

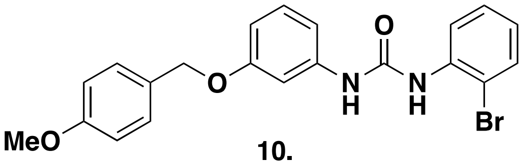

10.

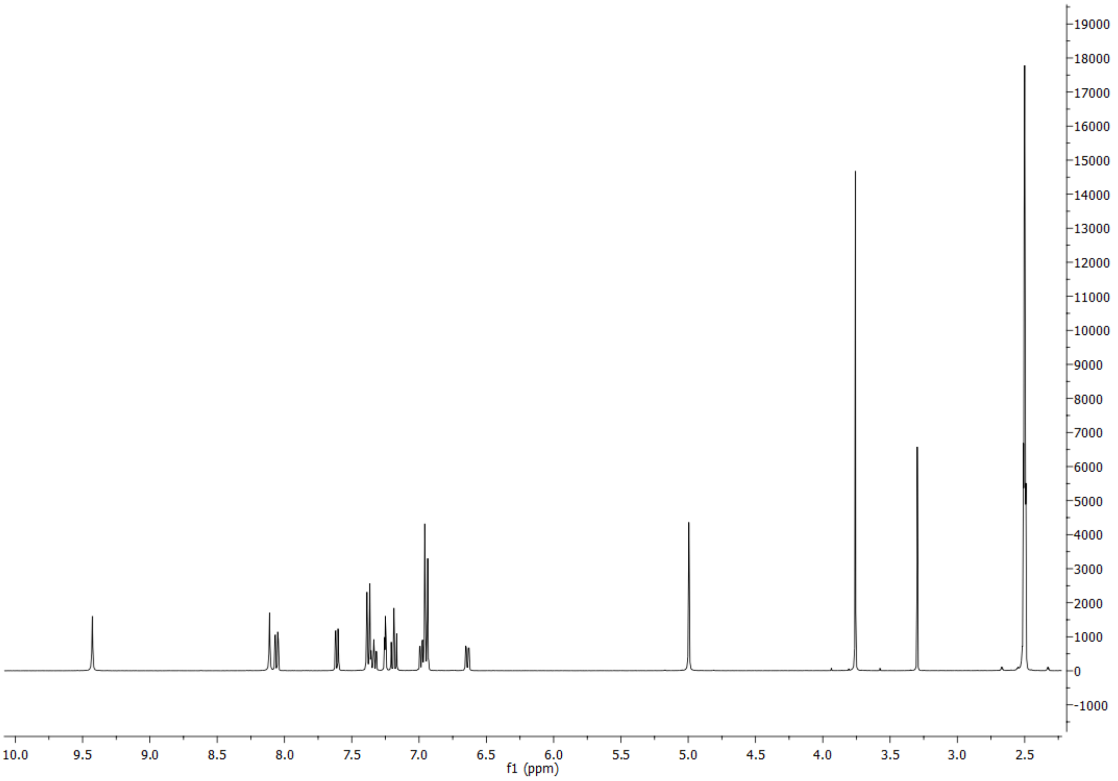

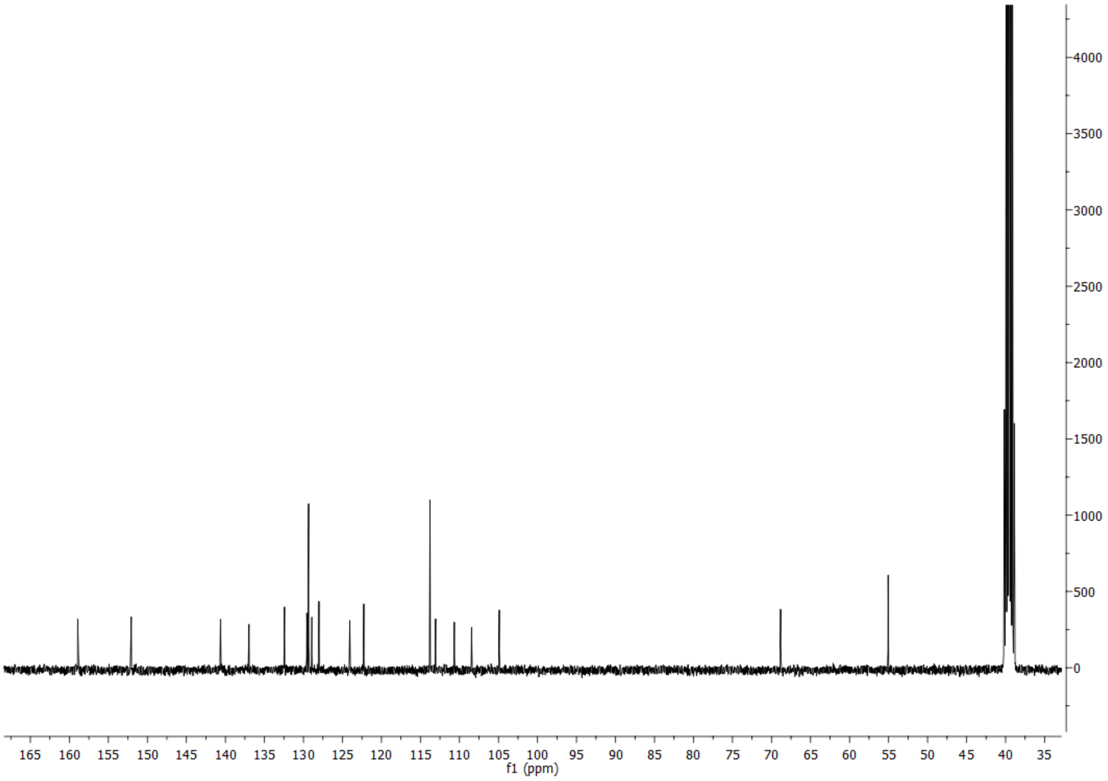

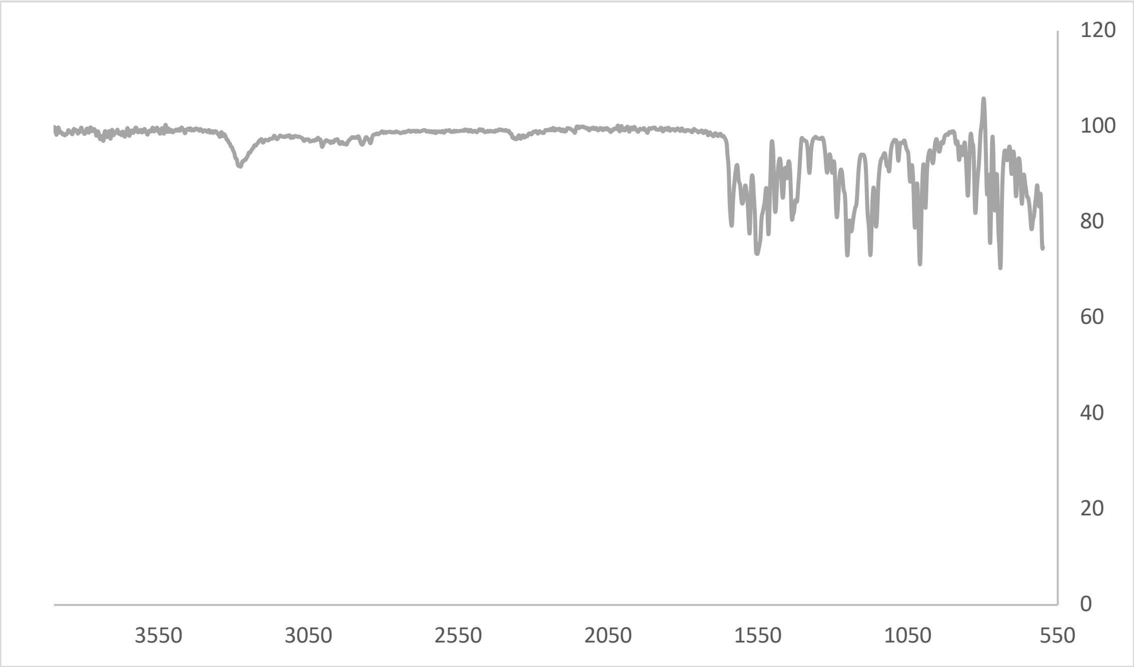
120
100
80
60
40
20
0
3550
3050
2550
2050
1550
1050
550

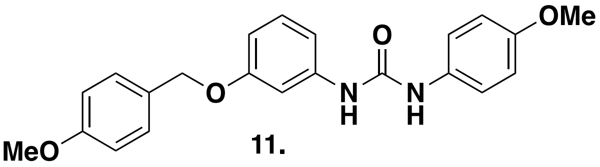

11.

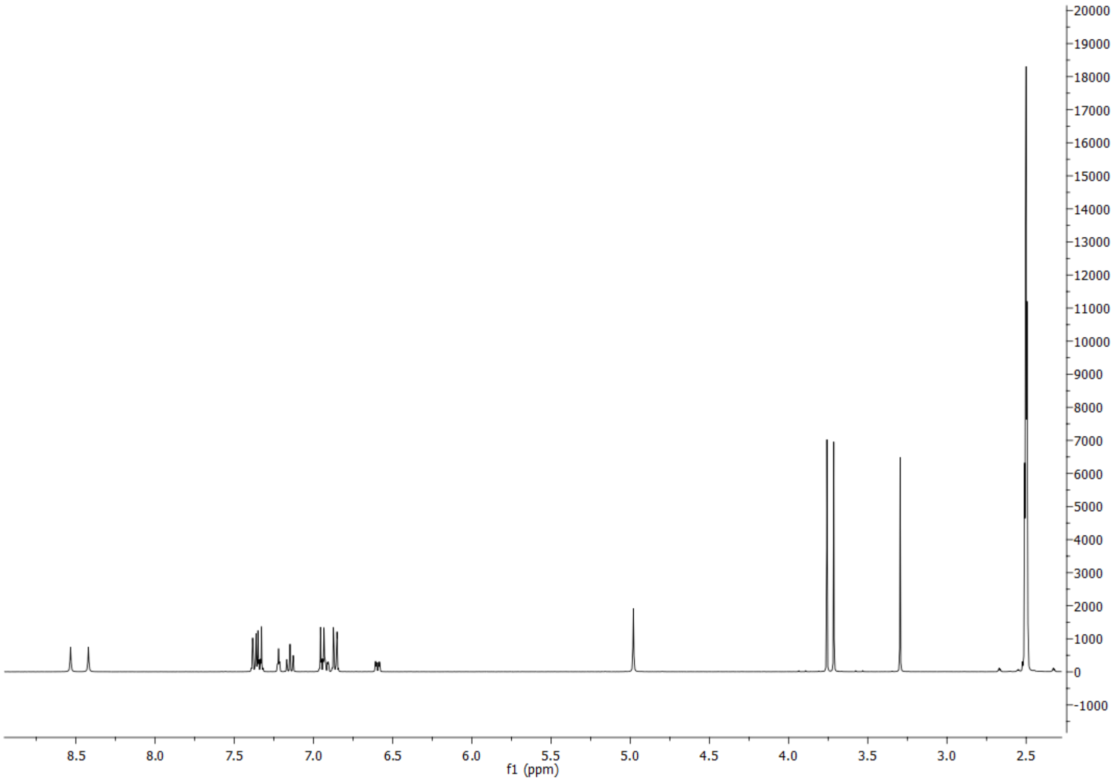

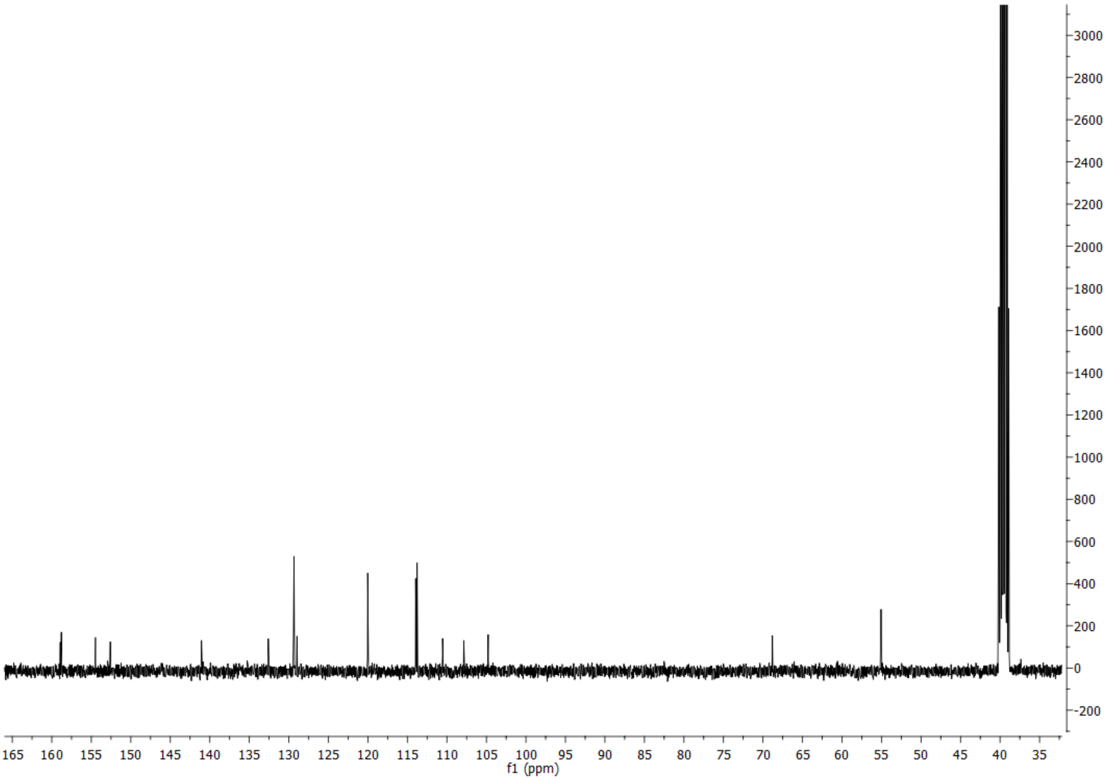

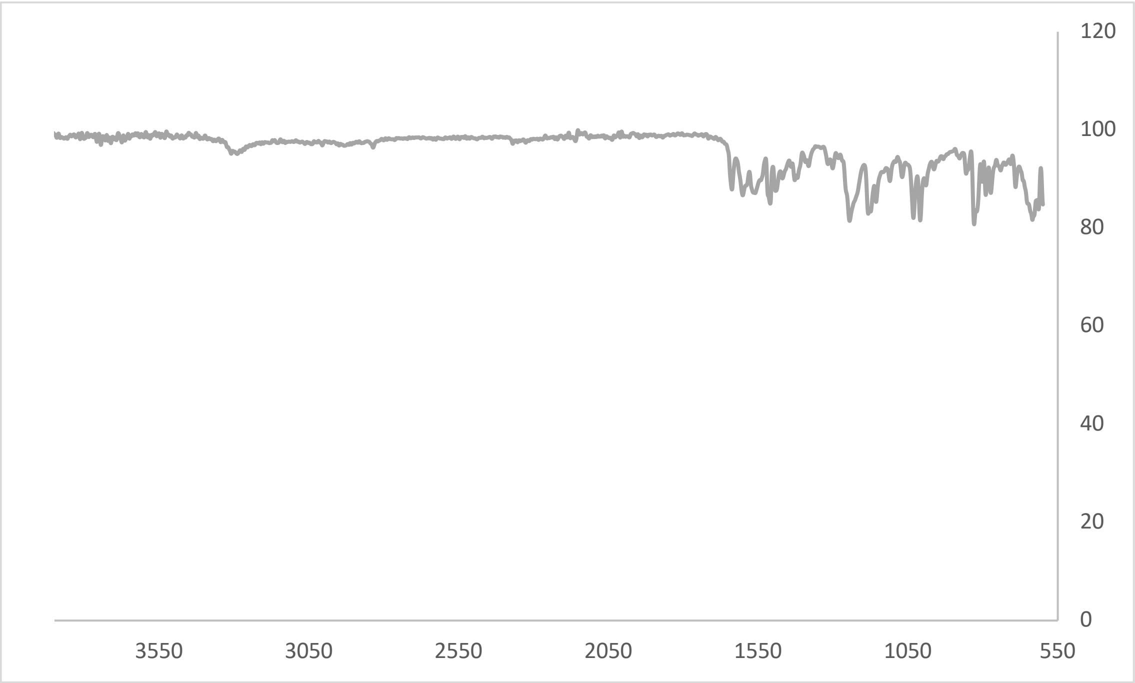
120
100
80
60
40
20
0
3550
3050
2550
2050
1550
1050
550

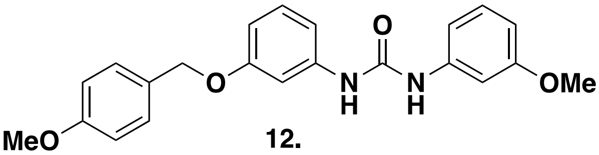
O
N
H
N
H
OMe
O
MeO

12.

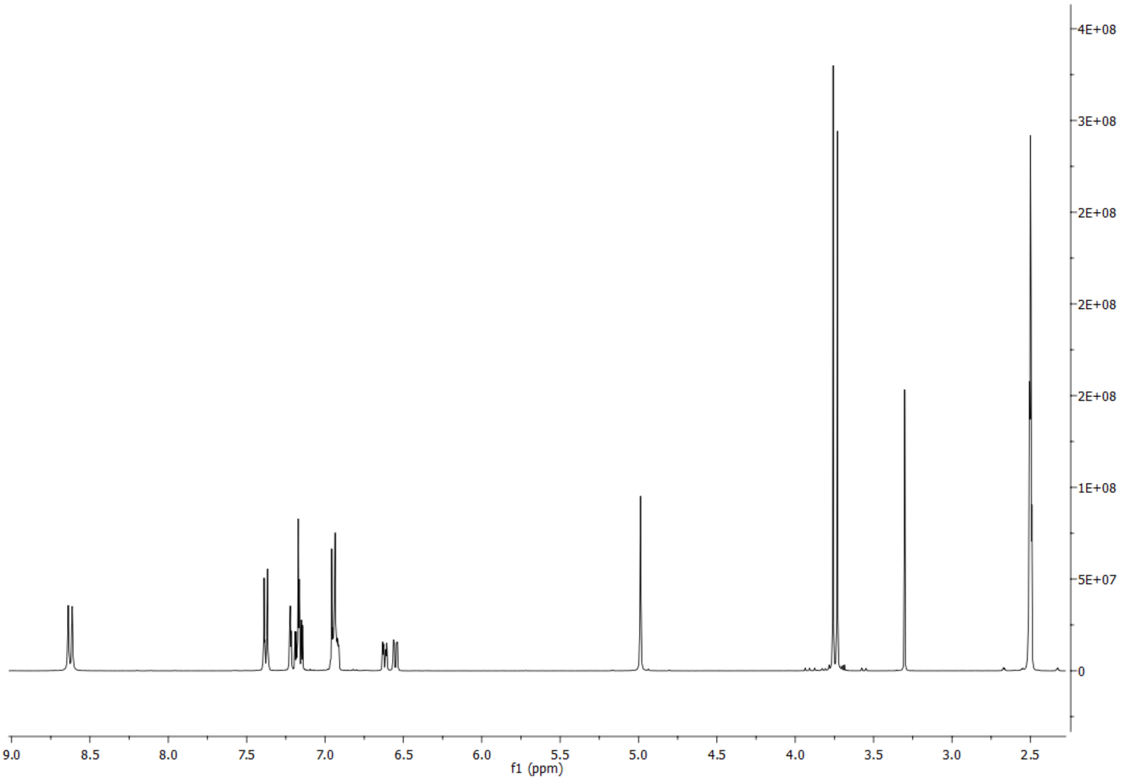
4E+08
3E+08
2E+08
2E+08
2E+08
1E+08
5E+07
0
9.0 8.5 8.0 7.5 7.0 6.5 6.0 5.5 5.0 4.5 4.0 3.5 3.0 2.5
f1 (ppm)

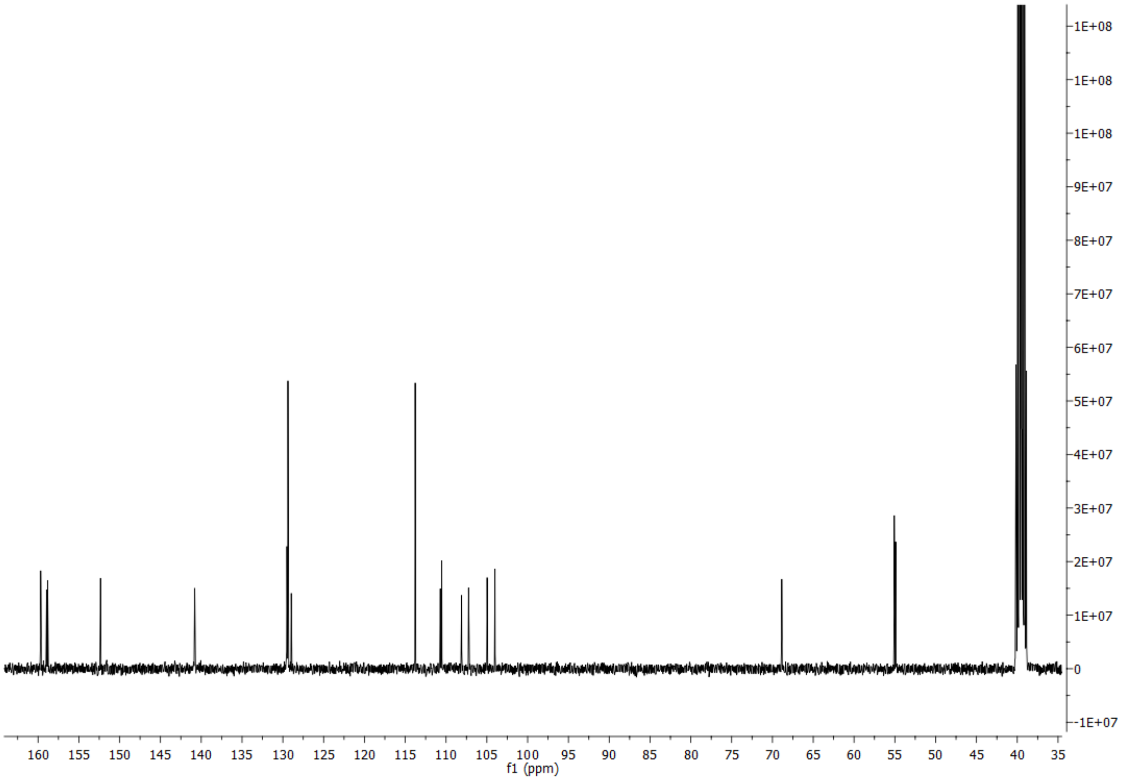
1E+08
1E+08
1E+08
9E+07
8E+07
7E+07
6E+07
5E+07
4E+07
3E+07
2E+07
1E+07
0
-1E+07
160 155 150 145 140 135 130 125 120 115 110 105 100 95 90 85 80 75 70 65 60 55 50 45 40 35
f1 (ppm)

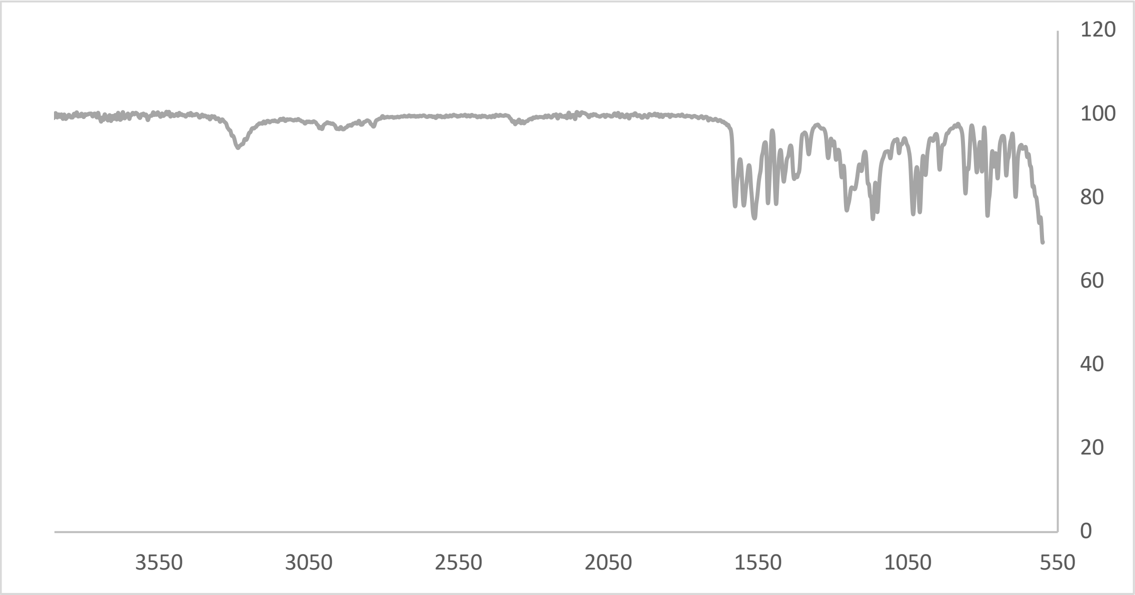
120
100
80
60
40
20
0
3550
3050
2550
2050
1550
1050
550

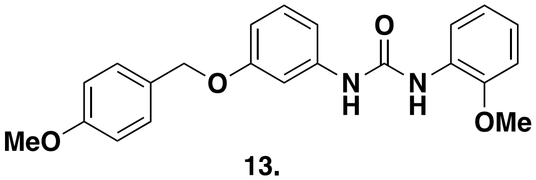

13.

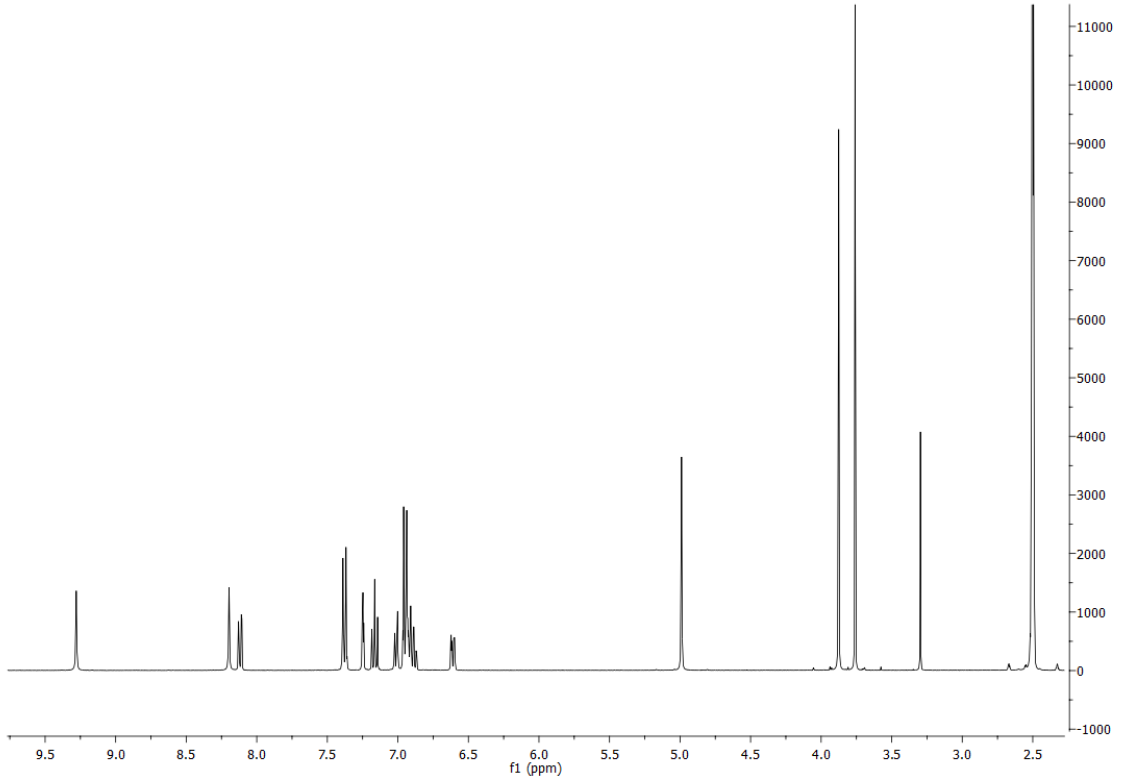

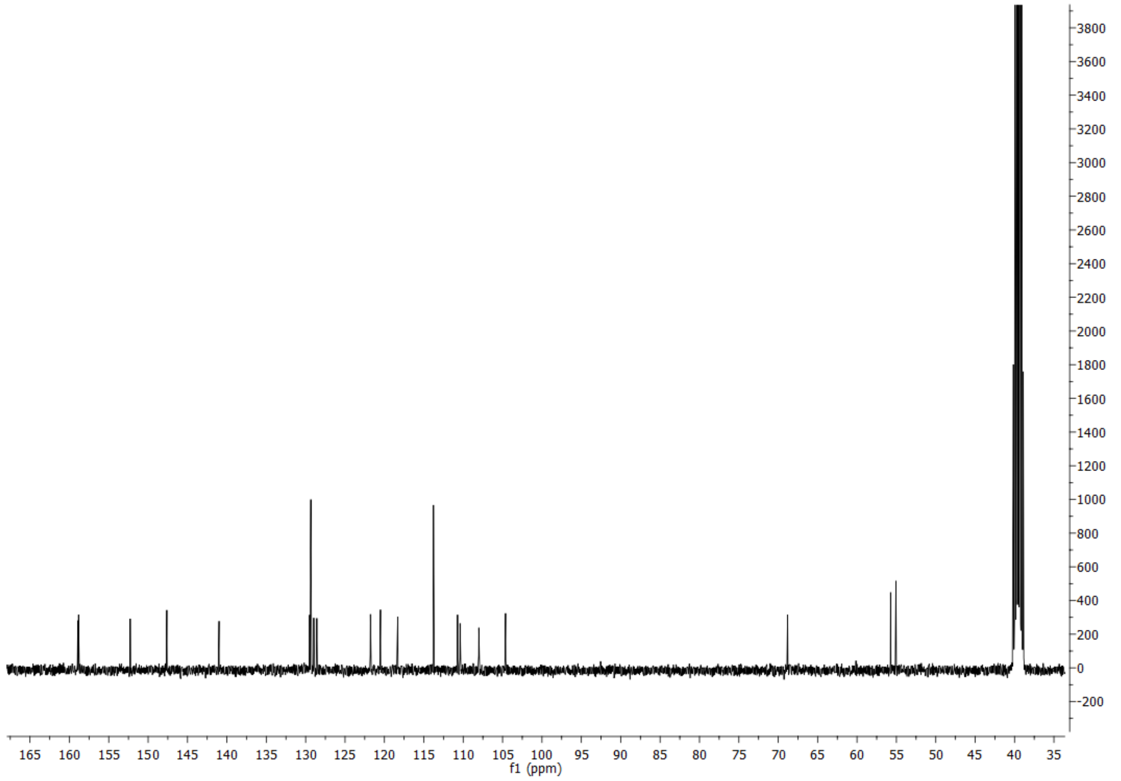

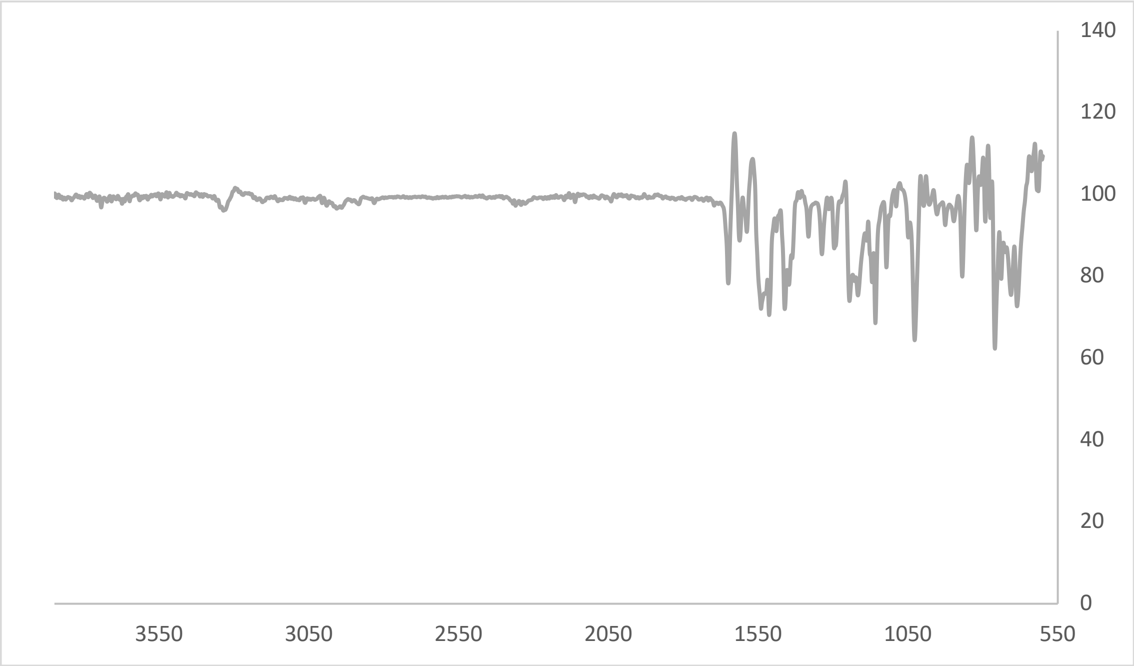
140
120
100
80
60
40
20
0
3550
3050
2550
2050
1550
1050
550

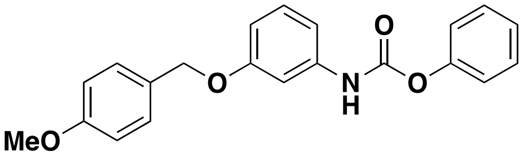

14.

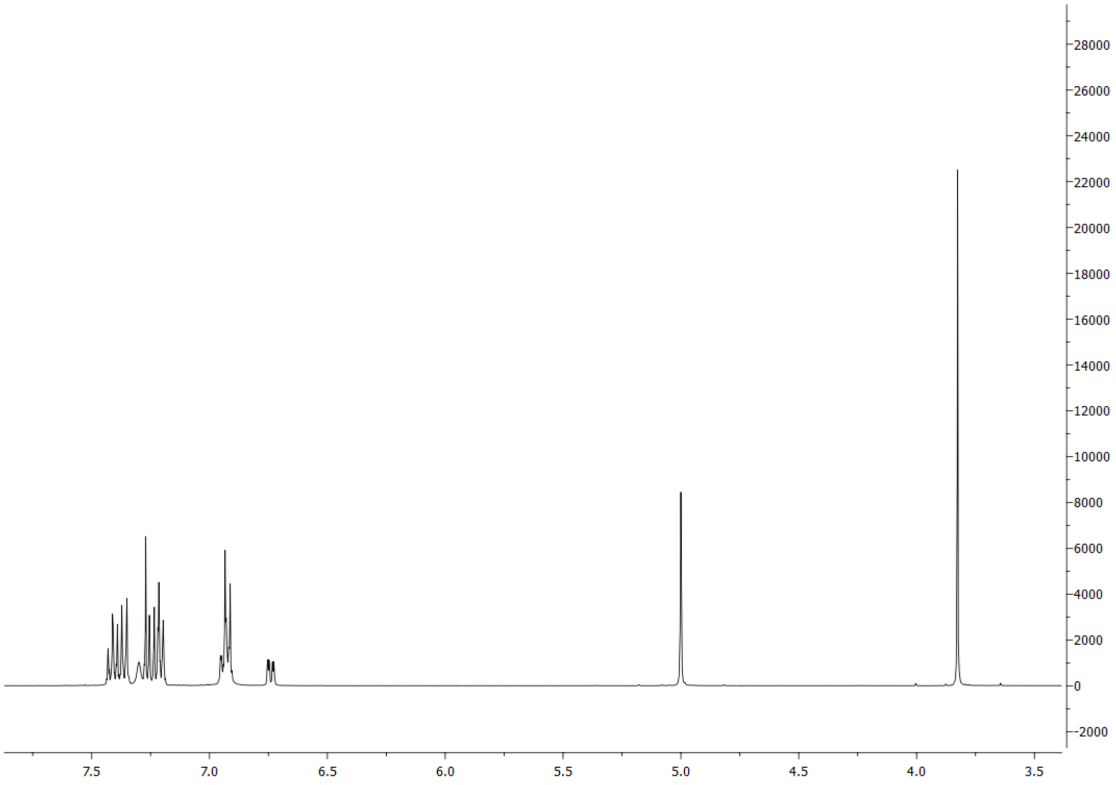

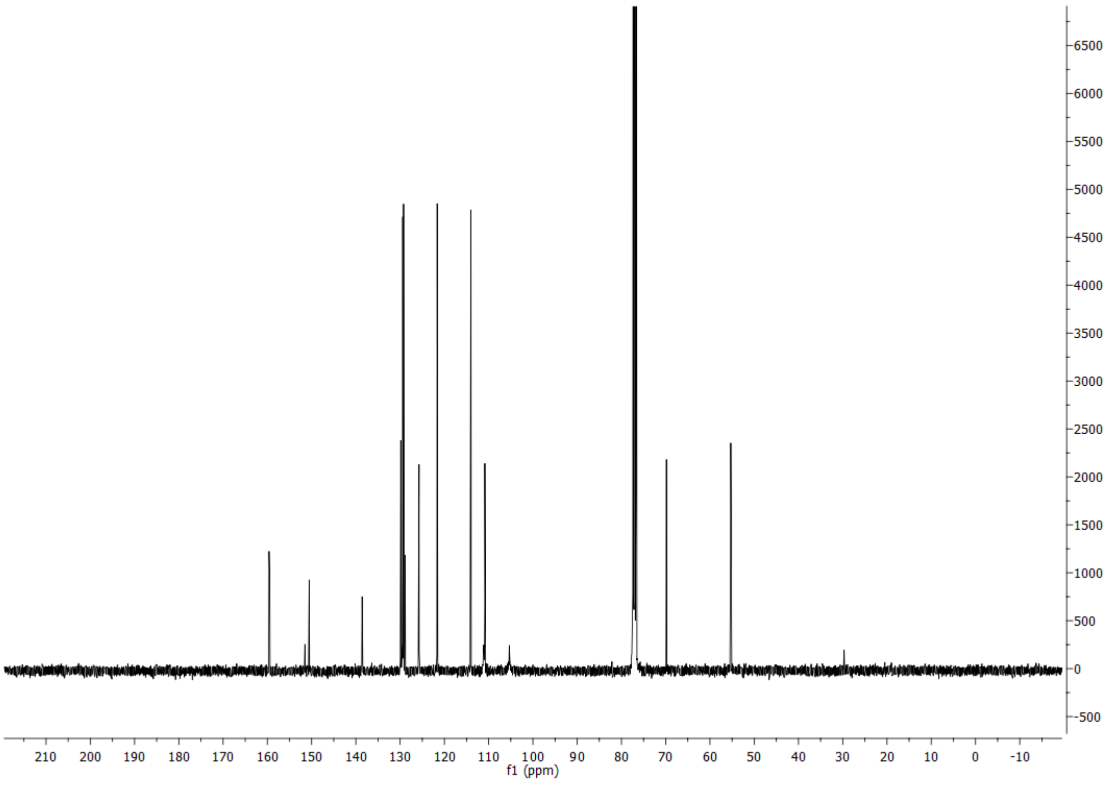

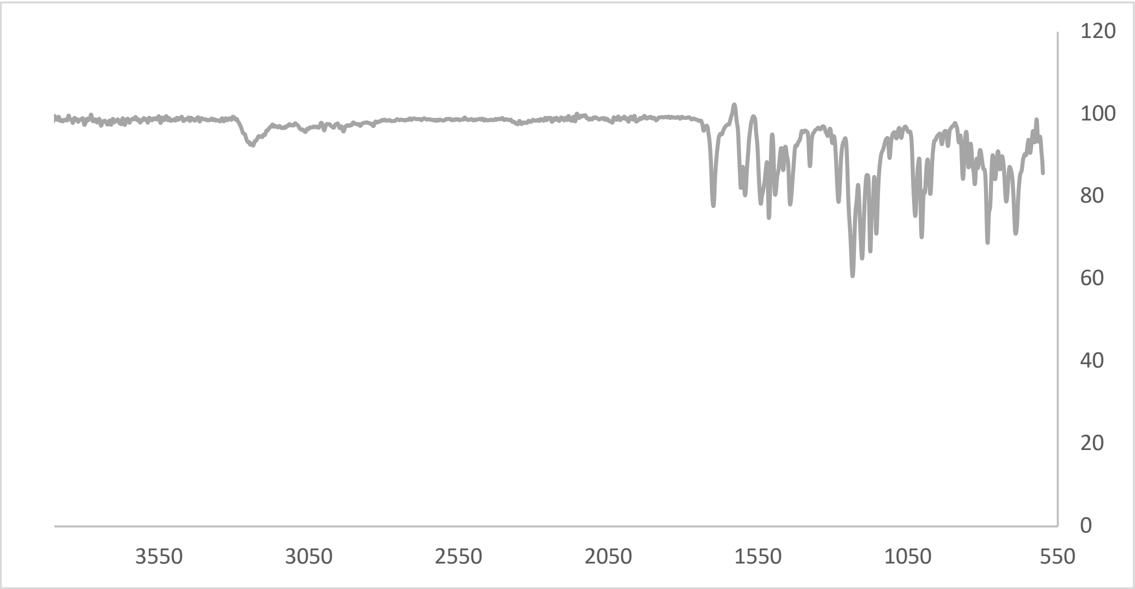
120
100
80
60
40
20
0
3550
3050
2550
2050
1550
1050
550
